# Preclinical Characterization of XB010: A Novel Antibody–Drug Conjugate for the Treatment of Solid Tumors that Targets Tumor-Associated Antigen 5T4

**DOI:** 10.1158/1535-7163.MCT-24-1014

**Published:** 2025-08-21

**Authors:** Brian A. Mendelsohn, Kathleen R. Gogas, Jeffrey N. Higaki, Willy A. Solis, Inna Vainshtein, Jackie Cheng, Minjong Park, Marlene A. Hennessy, Christine M. Janson, Yutaka Matsuda, Robyn M. Barfield, Penelope M. Drake, Stepan Chuprakov, Colin L. Hickle, Tom Linz, Maxine Bauzon, Dominick Y. Yeo, Fangjiu Zhang, Ayodele O. Ogunkoya, Seema Kantak

**Affiliations:** 1 Discovery Biotherapeutics, Exelixis, Inc., Alameda, California.; 2 Toxicology, Exelixis, Inc., Alameda, California.; 3 Clinical Pharmacology and Pharmacometrics, Exelixis, Inc., Alameda, California.; 4 Catalent Pharma Solutions, Emeryville, California.

## Abstract

The oncofetal antigen 5T4 is expressed in many solid tumors, making it an attractive antitumor target. XB010 is a novel, 5T4-targeted, antibody–drug conjugate developed using the SMARTag platform to optimize tolerability. We describe the development, design, and preclinical characterization of XB010. *In vitro* and *in vivo* efficacy of XB010 was assessed in cell-derived xenograft breast cancer cell lines (MCF-7 and MDA-MB-468) and in patient-derived xenograft tumor models (squamous cell carcinoma of the head and neck, non–small cell lung cancer, and breast cancer). Additionally, the *in vivo* combinatorial efficacy of XB010 + anti–PD-1 antibody was assessed in an MC38-h5T4 syngeneic colon cancer xenograft model. The toxicity profile of XB010 was evaluated in both Sprague–Dawley rats and cynomolgus monkeys. XB010 demonstrated *in vitro* cytotoxic effects with sub-nanomolar potency in the MCF-7 and MDA-MB-468 breast cancer cell lines and *in vivo* tumor growth inhibition (80%–99%) compared with vehicle-treated animals in xenograft and patient-derived xenograft models at doses of 5 to 10 mg/kg XB010. In the syngeneic MC38-h5T4–expressing colon cancer xenograft model, XB010 + anti–PD-1 showed improved efficacy compared with either agent administered alone. XB010 safety assessments demonstrated tolerability of doses up to 60 mg/kg in rats and up to 25 mg/kg in nonhuman primates. XB010 is a novel anti-5T4 antibody–drug conjugate that exhibits potent antitumor activity, inhibiting cancer cell growth *in vitro* and tumor growth in various *in vivo* models, with an acceptable toxicity profile. These findings support the evaluation of XB010 in clinical studies.

## Graphical Abstract

**Figure:**
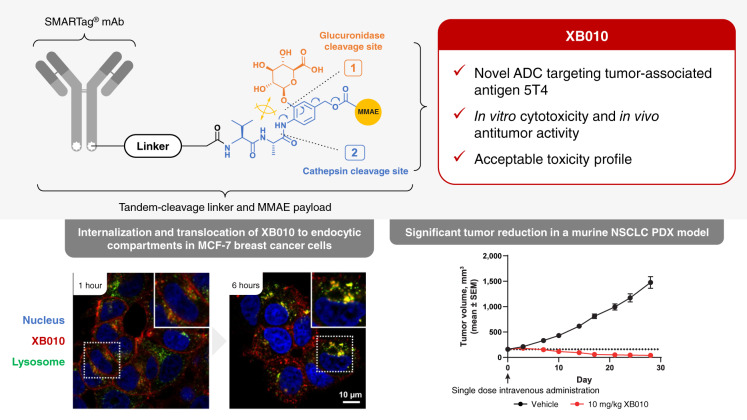

## Introduction

The oncofetal antigen 5T4 (also known as trophoblast glycoprotein or Wnt-activated inhibitory factor 1) is a 72 kDa *N*-glycosylated member of the leucine-rich repeat family of proteins that is associated with cells undergoing epithelial–mesenchymal transition, either during embryonic stem cell development or during epithelial tumor metastasis, via the regulation of signaling pathways that govern cell motility and mobilization (e.g., the Wnt/β-catenin pathway; refs. 1–3). Oncofetal antigens are proteins that are highly expressed in fetal tissues and tumors; however, they are only expressed in low levels in adult tissues (3, 4), thereby making them attractive candidates for anticancer therapies. In particular, 5T4 has garnered interest in recent years as a promising therapeutic target in many solid tumor types as it is overexpressed—frequently at high levels—in a wide range of solid tumors [e.g., breast, cervical, colorectal, ovarian, pancreatic, prostate, and renal cancers, and squamous cell cancer of the head and neck (SSCHN), mesothelioma, and non–small cell lung cancer (NSCLC)] but shows limited expression in normal adult tissues (3, 5). In addition, 5T4 expression has been associated with the survival of putative cancer stem cells, which function as the cellular drivers of tumors and contribute to tumorigenesis, eventually resulting in relapse after standard anticancer therapy (5). 5T4 expression has also been associated with worsening clinical outcomes in various cancer types, including NSCLC, SSCHN, and gastric, breast, pancreatic, and ovarian cancers (2, 3, 5–8). As such, targeted treatments against 5T4 may provide a new therapeutic approach for a wide variety of solid tumor types.

Antibody–drug conjugates (ADC), which comprise an antibody (directed against a specific cellular target), a chemical linker, and a cytotoxic payload that is released upon internalization by a target cell, have become a mainstay of targeted treatments for many solid and hematologic cancers (9, 10). However, one drawback with ADCs that have cleavable linkers, or less stable conjugation chemistries, is the potential for systemic payload shedding prior to tumor targeting and internalization (11). If an early, systemic release of the payload occurs, this not only reduces the potency of the remaining circulating ADC but can also lead to off-target, potentially dose-limiting toxicities (11). In addition, conventional conjugation of the linker–payload at lysine or cysteine residues on the antibody can lead to a degree of random conjugation, resulting in a complex heterogeneous mix of species with variable drug-to-antibody ratios (DAR; refs. 12, 13). This, in turn, can lead to rapid ADC clearance from circulation, a narrow therapeutic window, and poor ADC stability (13).

Recent developments in conjugation and linker technology design have attempted to address these issues by (i) enabling site-specific conjugation, to control the heterogeneity and the DAR, and (ii) improving the stability of the ADC in circulation, thereby reducing the level of free payload and minimizing off-target toxicities. One such approach is the SMARTag platform, which introduces bioorthogonal aldehyde handles at specific sites on the antibody via oxidation of cysteine residues (14). Site-specific conjugation of a payload–linker via a physiologically stable covalent C–C bond can then be performed using aldehyde-specific hydrazino-iso-Pictet-Spengler ligation chemistry (9, 12, 14). When combined with a tandem-cleavage linker strategy, in which two sequential enzymatic cleavage events are required for payload release (9), ADCs with such a design are expected to have optimized plasma stability, potentially improving efficacy and toxicity profiles compared with conventional conjugation methods (9).

**Figure 1.**
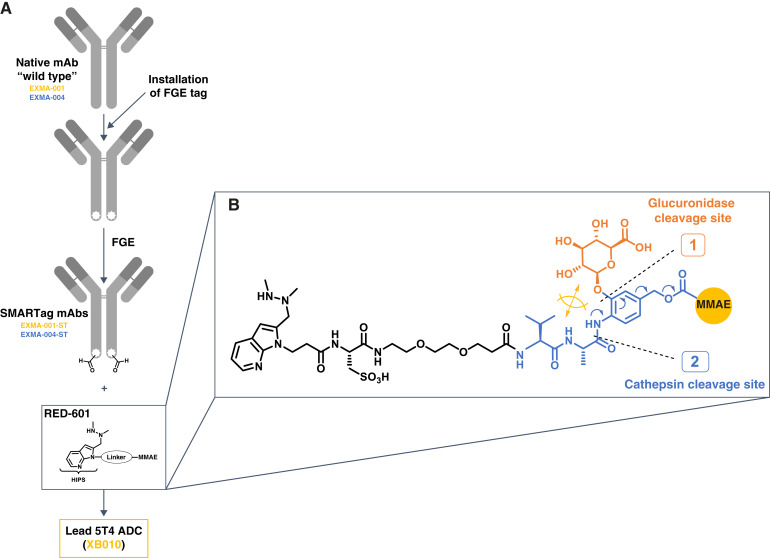
Development and design of the anti-5T4 ADC XB010: (**A**) preparation from lead mAbs and (**B**) structure of the tandem-cleavage linker–payload RED-601. FGE, formylglycine-generating enzyme.

XB010 is a novel 5T4-targeting ADC. It comprises (i) an anti-5T4 antibody selected for developability, stability, 5T4 binding, and internalization; (ii) a tandem-cleavage linker [RED-601; WO patent WO2023220620 (compound Vlb-82; ref. 15) and exemplified by Chuprakov and colleagues (9)]; and (iii) a microtubule polymerization inhibitor payload [monomethyl auristatin E (MMAE); ref. 16] conjugated using site-specific SMARTag technology (Fig. 1). These properties are designed to improve stability, efficacy, and tolerability (9, 17–22). Here, we describe the development, design, and preclinical characterization of XB010, including both *in vitro* and *in vivo* efficacy and toxicology.

## Materials and Methods

### Cell lines

MCF-7 and MDA-MB-468 cells (human female breast cancer lines that express 5T4) were acquired from the ATCC. Both cell lines were confirmed to be free of *Mycoplasma* and were authenticated using short tandem repeat profiling by ATCC prior to storage and cell line expansion. For the *in vitro* cytotoxicity studies, MCF-7 and MDA-MB-468 cells were acquired in 2013 and 2016, respectively; both cell lines were tested for *Mycoplasma* using the MycoAlert assay kit (Lonza, cat. #LT07-381) and used within 6 months of resuscitation. No additional *Mycoplasma* testing or authentication was conducted. Cells were maintained at 5% CO_2_ at 37°C and did not exceed 18 passages in culture.

### Size exclusion high-performance liquid chromatography analysis

Size exclusion high-performance liquid chromatography analysis of potential lead anti-5T4 mAbs was performed using a 7.8 mm (inner diameter) × 30 cm TSKgel G3000SWXL column (Tosoh Bioscience LLC, PN 08541) on an Agilent 1100 high-performance liquid chromatography (HPLC) system. Antibodies were normalized to 1 mg/mL concentration in Dulbecco’s PBS (pH 7.4, without Ca^2+^/Mg^2+^) and clarified via centrifugation to pellet particulates while still retaining soluble aggregates. For each sample, 10 μL was loaded to the column, isocratically eluted at 1.0 mL/minute over 20 minutes, and the absorbance was monitored at 280 nm. Chromatographic peaks were integrated to determine the percent homogeneity and retention time. Data analysis was performed using the Agilent ChemStation B.04.03.

### Hydrophobic interaction chromatography analysis

ADCs were examined by analytic hydrophobic interaction chromatography (Tosoh Bioscience LLC, cat. #14947) with mobile phase A [1.5 mol/L ammonium sulfate/25 mmol/L sodium phosphate (pH 7.0)] and mobile phase B [MPB; 25% isopropanol and 18.75 mmol/L sodium phosphate (pH 7.0); ref. 23].

### Enzymatic cleavage assay

To confirm the composition of the XB010 payload released via tandem enzymatic cleavage, β-glucuronidase (40 ng/μL, Sigma-Aldrich, cat. #3707580001) and human cathepsin B (20 ng/μL, Sigma-Aldrich, cat. #219362-50UGCN) were added to a solution of XB010 (1 mg/mL) in 30 μL of 2-morpholinoethanesulphonic acid buffer (10 mmol/L 2-morpholinoethanesulphonic acid-Na and 40 μmol/L dithiothreitol, pH 5.5) and incubated at 37°C for 6 hours. After quenching the reaction using Halt protease inhibitor cocktail (Thermo Fisher Scientific, cat. #78429), the reaction mixture was analyzed by reversed-phase HPLC (RP-HPLC) without purification.

### RP-HPLC analysis

RP-HPLC analysis was used to confirm release of the free MMAE payload. Samples [XB010, digested XB010, and MMAE payload (purchased from MedChemExpress, cat. #HY-15162, CAS: 474645-27-7)] were analyzed using an Agilent 1260 HPLC system equipped with an XBridge Shield RP 18, 4.6 × 150 mm, and 3.5 μm (Waters Corp.). The operational conditions were set as follows: a flow rate of 1.0 mL/minute at 50°C, with mobile phase A comprising 0.1% trifluoroacetic acid in water and MPB containing 0.1% trifluoroacetic acid in acetonitrile. Absorbance was monitored at 280 nm using a reference wavelength of 450 nm. A volume of 20 μL of the sample was injected into the system for each analysis. The elution profile included a 30-minute linear gradient from 1% to 30% MPB, followed by a 5-minute wash with 99% MPB and then a final 2-minute re-equilibration at 1% MPB.

### Internalization and localization of XB010

To determine the internalization properties of XB010, the endpoint internalization assay was employed. MCF-7 cells were incubated with either XB010, the mAb component of XB010 with SMARTag (EXMA-001-ST), or control (isotype-matched ADC) and incubated on ice for 30 minutes to label the surface 5T4 receptors. The cells were spun down at 4°C to remove any unbound antibodies, washed twice with cold PBS buffer, resuspended in fresh medium, and transferred to an incubator at 37°C to induce internalization for 20, 40, 60, 120, or 240 minutes. The cells were then transferred, for 20 minutes, to new plates containing cold 4% paraformaldehyde to stop internalization. Next, the cells were spun down, removed from fixation buffer, and washed twice with PBS before being stained on ice with 100 nmol/L of an Alexa Fluor 647–labeled goat anti–human IgG secondary antibody (Thermo Fisher Scientific, cat. #A48279) to detect surface-bound antibodies; they were then washed. Antibody internalization was determined in an indirect way by the decreased amounts of surface-bound antibodies at each timepoint compared with the respective surface-bound levels at the start of internalization. Analysis of surface-bound antibodies was performed using flow cytometry (CytoFlex Flow Cytometer, Beckman Coulter).

To measure trafficking of XB010 to endocytic compartments, MCF-7 cells prepared in a 96-well plate were treated with either XB010, EXMA-001-ST, or control that had been previously labeled with Incucyte Fabfluor-pH Orange Dye (working concentrations up to 4 μg/mL, cat. #4812; prepared per the manufacturer’s instructions). The 96-well plate was then immediately placed into the IncuCyte S3 Live-Cell Analysis System and imaged either in phase (to view cells) or under fluorescence (to view antibody internalization) every 30 minutes for up to 48 hours. Quantitative analysis of internalization [total red object integrated intensity (Red Calibrated Unit × μm^2^/well)] and cell confluency (percentage of the image area that is occupied by the cell) was performed using the IncuCyte Software system.

To evaluate colocalization of XB010 with lysosomal-associated membrane protein 1 (LAMP-1)–containing endocytic compartments, MCF-7 cells were incubated with XB010 (2 μg/mL) at 4°C for 30 minutes, unbound antibodies were washed away, and internalization was induced by incubation at 37°C with CO_2_ for 1 and 6 hours. Cells were then fixed with 4% paraformaldehyde, permeabilized, and incubated with mouse anti–human CD107a (LAMP-1) antibody (clone H4A3, BioLegend, cat. #328602). XB010 was detected using an Alexa Fluor 647–labeled goat anti–human IgG secondary antibody (Thermo Fisher Scientific, cat. #A48279) and LAMP-1 was detected using an Alexa Fluor Plus 488–labeled donkey anti–mouse IgG secondary antibody (Thermo Fisher Scientific, cat. #A32766). Cells were imaged using a spinning disk confocal microscope and colocalization was analyzed using Imaris AI Microscopy Image Analysis Software (Imaris) for Pearson correlation coefficient.

### *In vitro* cytotoxicity

To assess the *in vitro* cytotoxicity of XB010, eight concentrations (3 pmol/L to 50 nmol/L) were applied to breast cancer cell lines expressing the 5T4 antigen (MDA-MB-468 and MCF-7). Cell viability was measured after 120 hours using the CellTiter-Glo luminescent assay (Promega, cat. #G7573).

### *In vivo* efficacy of XB010

All *in vivo* studies were performed at Association for Assessment and Accreditation of Laboratory Animal Care–accredited facilities under protocols approved by the respective Institutional Animal Care and Use Committee.

#### Cell-derived xenograft models

The *in vivo* efficacy of XB010 was evaluated in two murine xenograft models of breast cancer: MCF-7 [female athymic nude mice (10 per treatment group); Charles River Discovery Services] and MDA-MB-468 (female SCID beige mice; eight per treatment group; Charles River Discovery Services). For both models, mice were inoculated with either MCF-7 or MDA-MB-468 cells via subcutaneous flank injection and randomized to treatment groups once the tumor had reached 100 to 150 mm^3^ in size. After randomization, the mice received intravenous doses [either one dose on day 1 or two doses (one on day 1 and one on day 7) in the MCF-7 model; one dose on day 1 and day 4 in the MDA-MB-468 model]. Dose levels for the two MCF-7 studies were as follows: XB010 (0.5, 2.5, or 5 mg/kg in the single-dose regimen or 0.25, 1.25, or 2.5 mg/kg for each of the two doses in the two-dose once week × two regimen). For the MDA-MB-468 model, animals received 5 mg/kg doses on day 1 and day 4. In all studies, a similarly conjugated negative control (isotype ADC) matched to the highest XB010 dose being evaluated, positive control (5T4-targeting mafodotin ADC; ref. 24) matched to the highest XB010 dose being evaluated, or vehicle (PBS or 20 mmol/L sodium citrate/50 mmol/L sodium chloride) was included. For the MDA-MB-468 study, the mAb from XB010 (EXMA-001) was also included as a treatment group (5 mg/kg). Body weight and tumor size were measured twice weekly throughout the studies, and animals were euthanized when the tumor size exceeded a specific threshold (700 mm^3^ for MDA-MB-468; 1,000 mm^3^ for MCF-7) or when a >20% body weight loss occurred. The tumor growth inhibition percentage (%TGI) was assessed at day 84 for MDA-MB-468 and day 26/28 for MCF-7 (one- and two-dose models, respectively), which represents the last day when 80% of animals remain in the vehicle group for each study.

#### XB010 in combination with an anti–PD-1 agent

To evaluate the efficacy of XB010 in combination with an anti–PD-1 checkpoint inhibitor (CD279, clone RMP1-14 from Bio X Cell), female C57BL/6 mice were inoculated with MC38-h5T4 cells (a syngeneic colon cancer model) via subcutaneous flank injection and randomized to treatment groups once the tumor had reached 87 mm^2^ in size (10 per group; Crown Bioscience). In this study, XB010 or corresponding XB010 vehicle (PBS) was given as a single intravenous dose on day 0 of the study. Anti–PD-1 or corresponding vehicle (PBS) was administered intraperitoneally twice a week × 2 weeks (day 0, 3, 7, and 10). After randomization, the mice received one of the following dosing regimens: XB010 vehicle + anti–PD-1 vehicle, 5 mg/kg XB010 (single dose) + anti–PD-1 vehicle, XB010 vehicle +0.5 mg/kg anti–PD-1, XB010 vehicle +0.75 mg/kg anti–PD-1, 5 mg/kg XB010 (single dose) + 0.75 mg/kg anti–PD-1, or 5 mg/kg XB010 (single dose) + 0.5 mg/kg anti–PD-1. Tumor volumes were measured twice weekly (in two dimensions), and animals were euthanized when the tumor size measured 2,000 mm^3^, >20% body weight loss occurred, or they reached day 55 (from randomization). %TGI was assessed at day 18 (the last day all animals were on study).

#### Patient-derived xenograft models

Patient-derived xenograft (PDX) models (Crown Bioscience; SCCHN, NSCLC, and breast cancer) were selected based on *H*-scores for 5T4 expression (determined by IHC methods) between 100 and 200. Single intravenous doses of 10 mg/kg XB010 or vehicle were administered to female NOD/SCID mice or Balb/c nude mice (10 per group/study). The tumor volume was monitored twice weekly for at least 28 days after treatment. Animals were euthanized when the tumor reached >3,000 mm^3^ in size, >20% body weight loss occurred, or they reached the prespecified end of study. %TGI was assessed on the last day when 80% of animals remain in the vehicle group.

### Statistical analyses

For *in vitro* cytotoxicity assays, survival was calculated as the percentage of luminescence (lum) in experimental wells relative to the untreated control wells. The average luminescence of the untreated wells was considered 100% viability. Specifically, the formula for calculating the percentage viability of an individual experimental well was as follows: % viability = 100−(lum_avg untreated_−lum_experimental_)/lum_avg untreated_ × 100. Data were displayed graphically using GraphPad PRISM. To calculate absolute IC_50_, the normalized data were analyzed using a dose–response curve fitted using a non-linear regression model and a four-parameter dose response.

For the MCF-7 and MDA-MB-468 cell-derived xenograft (CDX) *in vivo* efficacy studies, statistical analyses were performed using GraphPad PRISM software. Statistical analyses for the PDX and CDX combination efficacy studies were performed in the R language and environment for statistical computing and graphics. Depending on the normality of *in vivo* efficacy data, either an ANOVA or a Kruskal–Wallis test was conducted for comparisons, with appropriate *post hoc* tests performed in cases in which there were three or more groups. For the PDX studies, pairwise comparisons were carried out using either the Mann–Whitney *t* test or Welch *t* test. For the MCF-7, MDA-MB-468, and PDX studies, %TGI was defined as (1−Ti/Ci)×100, in which Ti and Ci were the mean tumor volumes of the treatment and control groups, respectively (on a given day). For the combination CDX study in the MC38-h5T4 model, %TGI was defined as 1−(Vt/V0)/(Ct-C0) × 100, in which V was the treated tumor volume at time t or 0 and C was the control (vehicle) volume. The log-rank (Mantel–Cox) test was used for survival analysis. In all cases, *P* values of < 0.05 were considered statistically significant.

### Toxicity and toxicokinetics

The safety and toxicokinetic (TK) profiles of XB010 were evaluated in exploratory toxicology studies conducted by Association for Assessment and Accreditation of Laboratory Animal Care–accredited contract research organizations in accordance with their Institutional Animal Care and Use Committee guidelines.

An acute single-dose study was performed in rats (non–antigen-binding species) to characterize XB010 exposure and safety findings attributed to the MMAE payload as a result of *in vivo* XB010 clearance and catabolism. Doses were arbitrarily selected to characterize toxicity based on dose ranges that were tolerated in rats with other DAR2 site-specific MMAE platforms (25, 26). Female Sprague–Dawley rats were randomly assigned to four groups (five per group). A single intravenous bolus injection of vehicle (20 mmol/L sodium citrate/50 mmol/L sodium chloride) or XB010 (30, 60, and 90 mg/kg doses) was administered on day 1. Toxicity was assessed based on mortality, clinical observations, body weight, and clinical pathology. Depending on survival, animals were euthanized for anatomic pathology evaluation (gross and microscopic) on day 12 (or earlier in case of intolerability). Blood samples were collected at several timepoints for TK analysis. XB010 exposure was evaluated based on total antibody (unconjugated or conjugated to the payload) and total ADC (antibody conjugated to the payload) levels that were measured in plasma using two ELISAs in sandwich formats with anti-human antibody capture detection for total antibody and anti-human and anti-MMAE capture detection for ADC.

Because XB010 binds to human and nonhuman primate (NHP; cynomolgus monkeys) 5T4, NHPs were considered to be a pharmacologically relevant species for the evaluation of antigen-dependent and antigen-independent (payload-related) toxicity findings. Experimentally naïve female cynomolgus monkeys were randomly assigned to four groups (three per group). Doses were selected to characterize exposure and toxicity responses over a broad range. Considerations for dose selection for the NHP study were also based on results from the rat toxicology study (following determination of the MTD) as well as doses that were tolerated in NHP from other DAR2 site-specific MMAE platforms (25). Vehicle (20 mmol/L sodium citrate/50 mmol/L sodium chloride) or XB010 (1, 6, and 25 mg/kg) was administered intravenously on days 1 and 22. Toxicity was assessed based on clinical observations, body weight, physical examinations, ophthalmology, electrocardiograms, and clinical and anatomic pathology (gross and microscopic evaluation of tissues collected at necropsy on day 43). Blood samples were collected at several time points for TK analysis. XB010 exposure was evaluated based on total antibody (unconjugated or conjugated to the payload) and total ADC (antibody conjugated to the payload) and unconjugated payload (MMAE) levels. Total antibody and total ADC were measured in serum samples using two separate qualified ELISAs in sandwich formats. Antigen (human recombinant 5T4) capture and anti-human antibody detection were used for total antibody and antigen capture and anti-MMAE detection was used for total ADC. Unconjugated MMAE levels were measured in plasma samples using an LC/MS assay.

## Results

### Characterization of lead anti-5T4 mAbs

Biopanning of naïve human genetic libraries was conducted using the hu-5T4 ECD (S32-S355)-Avi tag, and 14 unique clones were identified and reformatted into IgG1. Of these, the mAbs EXMA-001 and EXMA-004 were identified as lead candidates for anti-5T4 ADC development. As well as demonstrating promising developability (e.g., a single monomeric peak observable on size exclusion HPLC; Figs S1A and S1B), stress testing showed that both the EXMA-001 and EXMA-004 mAbs were thermally stable (melting points of 86.4°C and 81.9°C, respectively; Supplementary Table S1) and that their antigen binding did not change after exposure to pH 5.5 conditions for 14 days, pH 8.4 for 14 days, or oxidative conditions (Supplementary Fig. S2). Both mAbs also bound with high affinity to recombinant human and cynomolgus monkey 5T4 (K_D_ of 8.8–32.5 nmol/L), had no complementarity-determining region sequence liabilities, and showed high levels of internalization in Hu-5T4-HEK293 and MCF-7 cells (Supplementary Table S1).

### Development and design of XB010

Anti-5T4 ADCs were prepared by first converting native EXMA-001 and EXMA-004 to aldehyde-containing SMARTag mAbs (EXMA-001-ST and EXMA-004-ST), which expressed well and retained their thermal stability (melting points of 86.0°C and 80.9°C, respectively). XB010 was then prepared by hydrazino-iso-Pictet-Spengler conjugation of EXMA-001-ST to RED-601 (a linker–payload containing the tandem-cleavage component and an MMAE payload; Fig. 1; Supplementary Fig. S3). A similar process was used with EXMA-004-ST to create a separate anti-5T4 ADC; however, XB010 is the focus of the present study. The antibody purification and ADC tangential flow filtration yields were 94% and 84%, respectively, and the aldehyde conjugation yield was 98%. XB010 retained the target binding affinity of the native mAb [K_D_ of 7.5 (residual SD 10.5) and 26.3 nmol/L (residual SD 7.3) for human and cynomolgus monkey 5T4, respectively; Supplementary Table S1] and displayed suitable chemistry, manufacturing, and controls properties. This conjugation approach enables tight control of the DAR as measured using peak areas from hydrophobic interaction chromatography (DAR 1.8; Supplementary Fig. S4).

### Enzymatic cleavage assay

After the treatment of XB010 with β-glucuronidase and cathepsin B (tandem enzymatic cleavage), release of the free MMAE payload was confirmed by RP-HPLC analysis (Supplementary Fig. S5).

### Internalization and localization of XB010

Flow cytometry and live cell image analysis of XB010 internalization by MCF-7 cells showed that XB010 was readily internalized (∼45%), with a half-life shorter than the native EXMA-001 mAb containing SMARTag (EXMA-001-ST; 14.3 vs. 15.5 minutes), whereas the isotype-matched ADC control showed little to no internalization (Supplementary Fig. S6A). Similarly, both XB010 and EXMA-001-ST translocated to endocytic compartments in MCF-7 cells, whereas the isotype-matched ADC did not (Supplementary Fig. S6B). XB010-treated MCF-7 cells also exhibited decreased cell proliferation, whereas both EXMA-001-ST–treated and isotype-matched ADC-treated cells continued to proliferate at a similar rate (Supplementary Fig. S6C). Lastly, XB010 showed significant colocalization with LAMP-1–containing endocytic compartments in MCF-7 cells after 6 hours of incubation (Supplementary Fig. S6D).

### *In vitro* cytotoxicity of XB010 in MDA-MB-468 and MCF-7 breast cancer cell lines

**Figure 2.**
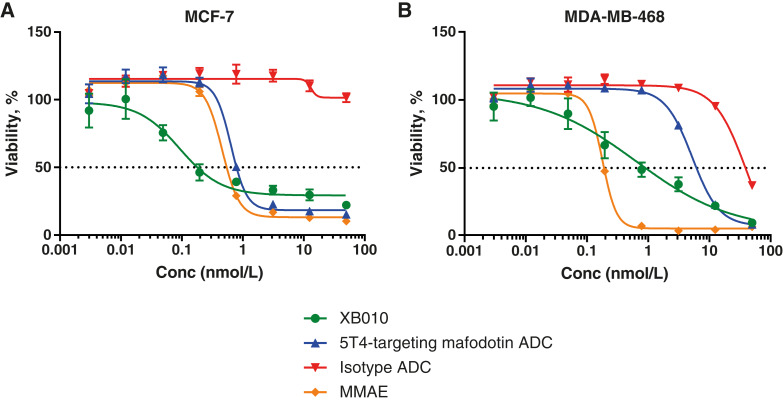
Cytotoxic activity of XB010 in (**A**) MCF-7 and (**B**) MDA-MB-468 breast cancer cell lines. XB010-mediated *in vitro* cytotoxicity was observed in both tumor cell lines that were used for *in vivo* xenograft studies. In both cell lines, XB010 demonstrated improved potency and similar maximal inhibition compared with the positive control (5T4-targeting mafodotin ADC). Conc, concentration.

XB010 had potent cytotoxic activity, with IC_50_ values of 0.896 nmol/L [95% confidence interval (CI), 0.53–1.5] and 0.185 nmol/L (95% CI, 0.10–0.33) and maximum inhibition values of 90.2% and 77.8% for MDA-MB-468 and MCF-7 cell lines, respectively (Fig. 2A and B). The 5T4-targeting mafodotin ADC had four- to seven-fold less potency than XB010, with IC_50_ values of 6.4 nmol/L (95% CI, 4.7–8.8) and 0.78 nmol/L (95% CI undefined) in MDA-MB-468 and MCF-7 cells, respectively (Fig. 2A and B). In contrast, an isotype-matched control ADC (e.g., a non–5T4-binding SMARTag IgG1 antibody conjugated to RED-601) had little to no effect on tumor cell viability, confirming that 5T4 binding is required for cytotoxicity.

### Tumor regression with XB010 in MCF7 and MDA-MB-468 breast cancer xenograft models

**Figure 3.**
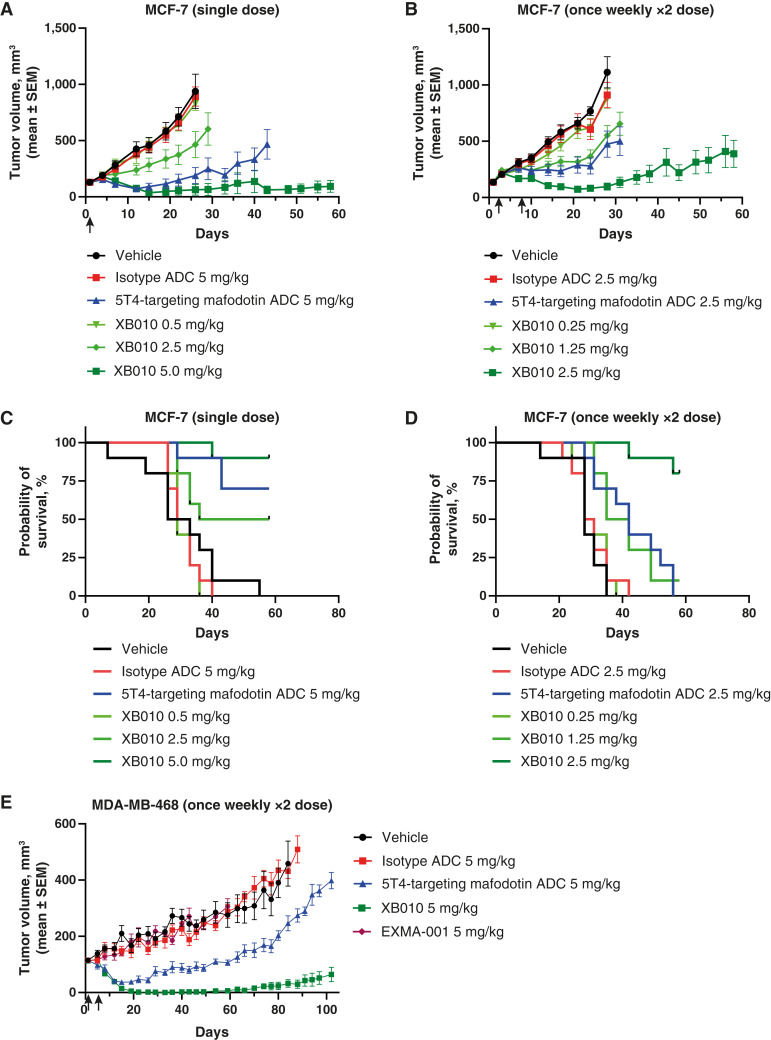
*In vivo* tumor growth inhibition and survival curves following intravenous administration of XB010 in (**A–D**) the MCF-7 xenograft model and *in vivo* tumor growth inhibition in the (**E**) MDA-MB-468 murine breast cancer xenograft model. Dose–response study with either (**A**) a single dose or (**B**) once weekly x2 dosing of XB010 in the MCF-7 model. Survival curves for (**C**) single dose and (**D**) once weekly x2 dose studies. XB010 led to dose-related tumor volume suppression and longer survival (right-shifted curves) compared with vehicle following either dosing regimen. As shown in (**E**), XB010 produced maximal and durable suppression starting at 20 days after dose in the MDA-MB-468 model. Survival curves were not generated for this study as only two of eight animals in the vehicle reached the prespecified survival outcome.

In an MCF-7 model, intravenous administration of 0.5, 2.5, or 5 mg/kg XB010 as a single dose or of 0.25, 1.25, or 2.5 mg/kg XB010 in a multi-dose once weekly × 2 (day 1 and 8) schedule resulted in dose-related tumor regression within the first 20 days and inhibited tumor growth up to end of the study (58 days; Fig. 3A and B). In the single-dose study (Fig. 3C; Supplementary Table S2), %TGI values versus vehicle increased with dose [11%, 50% (*P* < 0.05), and 93% (*P* < 0.0001) for 0.5, 2.5, and 5 mg/kg XB010, respectively], as did the number of complete responses (CR; defined as a tumor volume of 0 mm^3^ for 3 consecutive days as related to the total number of animals in each group: 0 of 10, two of 10, and six of 10, respectively). The median survival time for vehicle-treated animals was 29.5 days compared with 29, 47, and >58 days (last day of study) for animals treated with 0.5 mg/kg, 2.5 mg/kg, and 5 mg/kg XB010, respectively. The same trends were observed in the multi-dose study (Fig. 3D; Supplementary Table S3), with %TGI values of 21%, 50% (*P* < 0.001), and 91% (*P* < 0.001) and CRs of 0 of 10, one of 10, and three of 10 for once weekly × 2 doses of 0.25, 1.25, and 2.5 mg/kg XB010, respectively. The median survival time in the vehicle group was 28 days compared with 29.5, 38.5, and >58 days (last day of study) for the 0.25 mg/kg, 1.25 mg/kg, and 2.5 mg/kg XB010 groups, respectively. In addition, in both the single- and multi-dose MCF-7 studies, 5T4-targeting mafodotin ADC resulted in a lower %TGI and fewer CRs than the highest XB010 dose tested (Supplementary Table S3). The isotype-matched ADC control also showed no statistically significant antitumor activity in either xenograft study (%TGI of 4.9% and 18%, respectively, with no CRs in either study).

In the MDA-MB-468 model, intravenous administration of 5 mg/kg XB010 resulted in near complete tumor regression within 20 days and inhibited tumor growth for up to 100 days after dose (Fig. 3E). In addition, the %TGI value versus vehicle was 93% (*P* < 0.0001) and CRs were seen in eight of 10 animals (Supplementary Table S4). In contrast, the 5T4-targeting mafodotin ADC did not exhibit durable tumor control (i.e., tumor regrowth was apparent ∼14 days after the last dose) and had a lower %TGI (46%; *P* < 0.01) with two of 10 CRs (Supplementary Table S4). Neither the isotype-matched ADC (5 mg/kg) nor the native EXMA-001 mAb (5 mg/kg) showed antitumor activity (%TGI of 5.8% and 4.9%, respectively, with no CRs in either group; Supplementary Table S4).

### Combinatorial *in vivo* efficacy with XB010 plus an anti–PD-1 agent in the murine h5T4 syngeneic colon cancer model

**Figure 4.**
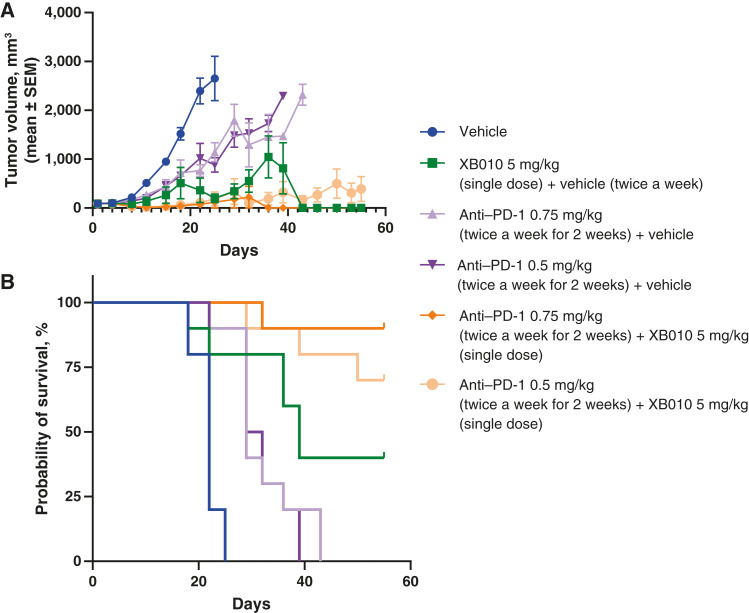
Combinatorial efficacy for XB010 and anti–PD-1 checkpoint inhibitor in a mouse syngeneic MC38-h5T4 model. Combination of XB010 and anti–PD-1 reagent enhanced antitumor activity (**A**) and improved survival (**B**) compared with either agent administered alone. Ten mice were included in each group.

A single dose of 5 mg/kg XB010 significantly inhibited tumor growth compared with control by day 18 (%TGI of 71%, *P* < 0.001; Fig. 4A; Supplementary Table S5), with CRs observed in four of 10 animals. In the XB010 + anti–PD-1 groups, the XB010-mediated inhibition of tumor growth was enhanced further, with %TGI of 97% and 96% for XB010 + 0.75 mg/kg anti–PD-1 and XB010 + 5 mg/kg anti–PD-1, respectively (both *P* < 0.001 vs. control) with CR rates of nine of 10 and five of 10, respectively; Supplementary Table S5). This enhanced tumor inhibition was not observed with the anti–PD-1 agent alone (0.75 mg/kg or 0.5 mg/kg dose), with %TGI values of 55% and 57% and CR rates of 0 of 10 and 0 of 10, respectively. The XB010 + anti–PD-1 treatment groups also showed the longest median survival (>55 days for XB010 + 0.75 mg/kg anti–PD-1 and XB010 + 0.5 mg/kg anti–PD-1), with other active treatment groups (vehicle +0.5 mg/kg anti–PD-1; vehicle +0.75 mg/kg anti–PD-1) having a median survival of 31 to 39 days. The control group had a median survival of 22 days (Fig. 4B).

### Durable tumor suppression with XB010 across multiple PDX models

**Figure 5.**
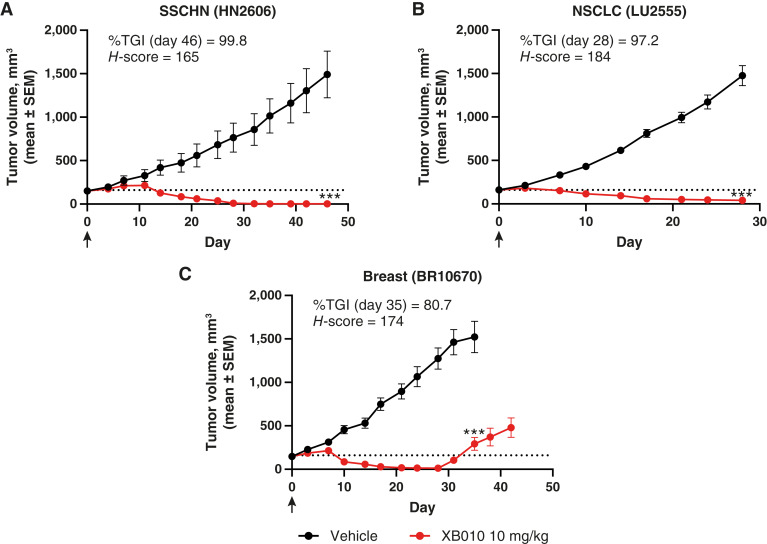
*In vivo* tumor growth inhibition in murine PDX models. Significant tumor reduction was observed following single-dose intravenous administration of XB010 (10 mg/kg) in murine PDX models of (**A**) SSCHN, (**B**), NSCLC, and (**C**) breast cancer.

A single intravenous dose of 10 mg/kg XB010 led to significant tumor reduction in 5T4-expressing PDX models across multiple indications, with %TGI values of 100%, 97%, and 81% for SSCHN (Fig. 5A), NSCLC (Fig. 5B), and breast cancer (Fig. 5C), respectively. XB010-treated animals showed tumor suppression and regression starting at ∼10 days after dose and lasting to at least 30 days after dose, with CRs in eight of 10, 0 of 10, and seven of 10 animals for the SCCHN, NSCLC, and breast cancer PDX models, respectively.

### TK profile of XB010

Following single intravenous doses of XB010 (30, 60, and 90 mg/kg) administered to female Sprague–Dawley rats, linear TK profiles were observed over 11 days. XB010 was stable and displayed nearly identical TK profiles for total antibody and total ADC (Supplementary Fig. S7; Supplementary Table S6). Following repeat intravenous doses of XB010 (1, 6, and 25 mg/kg) administered to NHPs on days 1 and 22, non-linear TK profiles were observed, indicating target-mediated drug disposition. XB010 was stable in NHPs, with nearly identical TK profiles for the total antibody and total ADC and low levels of unconjugated MMAE (<0.5 ng/mL MMAE at 25 mg/kg XB010; Supplementary Fig. S8). Immunogenicity assessment showed anti-drug antibodies (ADA) against XB010 in all animals in the 1-mg/kg group at timepoints near and/or at trough XB010 concentrations (starting on day 15). However, day 1 and day 22 area under the concentration–time curve exposures were similar, suggesting that ADA response had minimal effect on exposure. In the 6- or 25-mg/kg groups, ADAs against XB010 were not detected. Mean XB010 TK parameters in NHP are summarized in Table 1.

**Table 1.** Mean TK parameters for XB010 administered following repeat-dose administration to NHPs on day 1 and day 22.

​	Day 1				Day 22			
Dose (mg/kg)	C_max_ (μg/mL)	AUC_last_ (μg/mL·day)	CL (mL/day/kg)	t_1/2_ (days)	C_max_ (μg/mL)	AUC_last_ (μg/mL·day)	CL (mL/day/kg)	t_1/2_ (days)
1	26.8 (5.64)	27.4 (5.10)	37.2 (7.10)	0.975 (0.0473)	29.2 (1.29)	21.1 (12.6)	73.5 (66.0)	0.740 (0.439)
6	183 (41.5)	362 (49.5)	16.8 (2.29)	2.14 (0.138)	214 (16.7)	487 (61.1)	12.4 (1.66)	2.23 (0.4)
25	604 (66.1)	2,700 (322)	8.79 (1.33)	5.32 (1.11)	592 (23.2)	2,790 (478)	9.11 (1.44)	4.82 (0.848)

All data are presented as mean (SD).

AUC_last_, area under the concentration–time curve (from time 0 to last observation).

Abbreviations: AUC_last_, area under the concentration–time curve; CL, clearance; C_max_, maximum plasma concentration; t_1/2_, half-life.

### Tolerability and toxicity profile of XB010

In rats (non–antigen-binding species), single intravenous doses of XB010 were tolerated up to a dose of 60 mg/kg (MTD). At 90 mg/kg, there was XB010-related death or early euthanasia in two of five rats and overall toxicity (Supplementary Table S7). Notable clinical observations in XB010-treated rats (mainly in those receiving 60 or 90 mg/kg) included moderate-to-severe body weight loss at day 5 (which had generally recovered by day 12 at 60 mg/kg), increases in liver transaminases, bilirubin, and creatinine, and decreases in white blood cells, platelets, and red blood cell mass. Treatment-related histopathology analyses (at day 12) revealed dose-dependent increases in histopathology findings (both in incidence and severity). At doses of 60 mg/kg XB010 or above, these included lung inflammation (multifocal, chronic alveolar, and/or interstitial), kidney inflammation, bone marrow (hypocellularity), lymphoid tissue (atrophy and/or increased extramedullary hematopoiesis), and liver inflammation [extramedullary hematopoiesis, mitotic figures, inflammation, necrosis, and other changes in hepatocytes (vacuolation, karyomegaly, and hypertrophy)]. At the 90 mg/kg XB010 dose, additional findings were present in the heart (degeneration and necrosis or inflammation of ventricular myofiber) and gastrointestinal tract (multifocal epithelial necrosis).

In NHPs (antigen-binding species), XB010 was tolerated at doses of up to 25 mg/kg (Supplementary Table S8). All animals survived to their scheduled euthanasia on day 43 and there were no XB010-related effects on clinical observations, body weight, food consumption, ophthalmic observations, physical examination findings, or electrocardiograms in the NHPs administered up to 25 mg/kg XB010. Clinical pathology effects (e.g., changes in hematology and clinical chemistry parameters) were limited to animals administered 25 mg/kg XB010, and these were generally transient and returned to near-baseline levels by day 43. XB010-related histopathology findings were present in the thymus (decreased lymphocytes), bone marrow (increased cellularity), eye (apoptosis/necrosis and increased mitotic figures in corneal epithelium), lung (increased macrophage and mixed cell inflammation, fibroplasia, and respiratory epithelium hypertrophy), and stomach (apoptosis/necrosis and mixed-cell inflammation) of NHPs (primarily with 25 mg/kg XB010); these were all interpreted as antigen independent and considered non-adverse events because of their low severity and minimal impact on the health and well-being of the animals.

## Discussion

Preclinical characterization of the novel anti-5T4 ADC, XB010, and its native mAb (EXMA-001) demonstrated that both have high binding affinity to human and cynomolgus monkey 5T4, with XB010 displaying potent, target-mediated activity as a site-specific, tandem-cleavage, MMAE-containing ADC.

5T4 is an oncofetal glycoprotein that is a promising therapeutic target for many cancers, as it is expressed in a wide range of tumor types (including breast, cervical, ovarian, pancreatic, and renal cancers and NSCLC), but shows very limited expression in normal adult tissues (3, 27). As such, several anti-5T4 ADCs have been developed for the treatment of solid tumor types, including PF-06263507, an anti–5T4 humanized IgG1 conjugated to the microtubule disruptor monomethyl auristatin F (MMAF; ref. 24), and ASN004, an anti-5T4 single-chain Fv–Fc antibody conjugated to several auristatin F hydroxypropylamide payloads (27). In preclinical studies, ASN004 was shown to bind 5T4 with high affinity, displayed potent cytotoxic activity across a range of solid tumor cell lines, and led to durable regression in xenograft models of human lung, breast, cervical, and gastric tumors (26). Consistent with these data, in our studies, XB010 was shown to be internalized in 5T4-expressing tumor cells and produced near complete and durable tumor regression in both *in vivo* CDX (breast cancer) and PDX (SCCHN, NSCLC, and breast cancer) models. Taken together, these data support XB010 as a potential therapeutic candidate with utility across a range of solid tumor types.

Overall, there is growing evidence from preclinical studies and early clinical trials indicating that the combination of ADCs and established immunotherapies may enhance antitumor effects (10). Although the mechanisms behind this effect are unclear and likely diverse, it is believed to encompass the initiation of immunogenic cell death, dendritic cell maturation, enhancement of T-cell function, reinforcement of immunologic antitumor memory, and the expression of immunomodulatory proteins such as PD-1/PD-L1 (10). The combination of XB010 and an anti–PD-1 agent in a murine syngeneic colon cancer model enhanced tumor suppression compared with either compound administered alone, with mean tumor volumes reduced by ∼30-fold compared with controls, as well as %TGI of >95% and CRs in up to 90% of animals tested. These promising findings highlight the potential of XB010 as a combination partner in the treatment of cancers known to express 5T4.

Auristatin-based ADCs (including MMAF and MMAE) are associated with toxicities, including neutropenia, anemia, and thrombocytopenia, peripheral neuropathy, and skin and ocular toxicities (24, 28–32). For example, the phase I clinical trial of PF-06263507 (anti-5T4 MMAF ADC) was terminated because of dose-limiting ocular toxicity and limited tumor responses, with 38.5% of patients experiencing grade 1 to 2 treatment-related ocular adverse events (24, 32). XB010 is a 5T4-targeting ADC generated using the SMARTag platform and the RED-601 cleavable linker-MMAE, which creates site-specific conjugation and an ADC that requires tandem cleavage for payload release (9), thereby improving circulating ADC stability, reducing circulating unconjugated payload, and potentially reducing off-target toxicities. In light of this, the observed XB010-related safety findings at tolerated doses in rat and monkey were noted at much higher doses than those of stochastic MMAE conjugates. The observed XB010 toxicity findings in lymphohematopoietic organs, eye, and lung have also been seen with non–5T4-targeting site-specific MMAE conjugates in cynomolgus monkeys (25, 26), and they were interpreted to be independent of antigen.

In summary, XB010 is a novel anti-5T4 ADC that exhibits potent antitumor activity; it inhibits cancer cell growth *in vitro* and tumor growth in various *in vivo* murine xenograft models and PDX models. XB010 demonstrated acceptable safety profiles in nonclinical toxicology studies at tolerated doses. The XB010-related toxicity findings were interpreted to be antigen independent, reversible, and clinically monitorable. The efficacy data, and the potential of the tandem-cleavage linker to provide improved tolerability and exposure, suggest that XB010 has promising therapeutic potential in a range of solid tumor types and support the evaluation of XB010 in clinical studies as monotherapy and in combination with immunotherapies such as anti–PD-1 agents. XB010 is currently being evaluated in a phase I clinical trial (NCT06545331).

## Supplementary Material

Figure S1 Figure S1: SEC-HPLC chromatograms of EXMA-001 and EXMA-004. A single monomeric peak was observed for EXMA-001 and EXMA-004, indicating promising developability.

Figure S2 Figure S2: Antigen binding of EXMA-001 and EXMA-004. Oxidative stress testing of EXMA-001 and EXMA-004 showed no changes in antigen binding after treatment with hydrogen peroxide. By comparison, an oxidation event taking place in the complementarity-determining region of the antibody altered the binding of EXMA-005 to the target antigen, meaning that this candidate was not considered suitable for further development.

Figure S3 Figure S3: Structure of XB010. Chemical structure of the linker (RED-601) and monomethyl auristatin E payload, conjugated to the EXMA-001-ST monoclonal antibody using site-specific SMARTag technology.

Figure S4 Figure S4: HIC analysis of XB010. The DAR was confirmed as 1.8, as measured using peak areas from HIC versus a reference standard.

Figure S5 Figure S5: RP-HPLC analysis of XB010 following single and tandem enzymatic cleavage. Single and tandem enzymatic cleavage with β-glucuronidase and cathepsin B confirmed the release of unconjugated MMAE.

Figure S6 Figure S6: Translocation of XB010 to endocytic compartments in MCF-7 cells, MCF-7 cell confluency over time with XB010, and quantification of XB010 in MCF-7 LAMP-1 cell compartments after 1 and 6 hours of incubation.

Figure S7 Figure S7: TK profile of XB010 in rats. Following single IV doses of XB010 (30, 60, and 90 mg/kg) administered to female Sprague Dawley rats, linear TK profiles were observed over 11 days. Near-identical TK profiles were observed for the total antibody and total ADC, demonstrating the stability of XB010.

Figure S8 Figure S8: TK profile of XB010 in NHPs. Following repeat IV doses of XB010 (1, 6, and 25 mg/kg) administered to NHPs on days 1 and 22, non-linear TK profiles were observed, indicating target-mediated drug disposition. XB010 was stable in NHPs, with nearly identical TK profiles for the total antibody and total ADC, and low levels of unconjugated MMAE.

Table S1 Table S1: Summary of lead mAb and ADC properties.

Table S2 Table S2: Tumor growth inhibition, response rates, and survival following IV administration of XB010 and controls (single dose in MCF-7 xenograft model).

Table S3 Table S3: Tumor growth inhibition, response rates, and survival following IV administration of XB010 and controls (QWx2 dosing in MCF-7 xenograft model).

Table S4 Table S4: Tumor growth inhibition and response rates following IV administration of XB010 and controls (MDA-MB-468 xenograft model).

Table S5 Table S5: Tumor growth inhibition, response rates, and survival following IV administration of XB010 alone or in combination with anti-PD1 (h5T4-MC38 model).

Table S6 Table S6: Mean TK parameters for XB010 (total ADC) administered following single-dose administration to rats over an 11-day observation period.

Table S7 Table S7: Toxicity of XB010 (30, 60 and 90 mg/kg doses) in rats.

Table S8 Table S8: Toxicity of XB010 (1, 6 and 25 mg/kg doses) in NHPs.

## Data Availability

The data generated in this study are available within the article and its supplementary data files.

## References

[1] Nieto MA , Cano A. The epithelial–mesenchymal transition under control: global programs to regulate epithelial plasticity. Semin Cancer Biol 2012;22:361–8. 22613485 10.1016/j.semcancer.2012.05.003

[2] Zhao Y , Malinauskas T, Harlos K, Jones EY. Structural insights into the inhibition of Wnt signaling by cancer antigen 5T4/Wnt-activated inhibitory factor 1. Structure 2014;22:612–20. 24582434 10.1016/j.str.2014.01.009 PMC3988984

[3] Stern PL , Harrop R. 5T4 oncofoetal antigen: an attractive target for immune intervention in cancer. Cancer Immunol Immunother 2017;66:415–26. 27757559 10.1007/s00262-016-1917-3 PMC11029567

[4] Wepsic HT . Overview of oncofetal antigens in cancer. Ann Clin Lab Sci 1983;13:261–6. 6194734

[5] Harrop R , O’Neill E, Stern PL. Cancer stem cell mobilization and therapeutic targeting of the 5T4 oncofetal antigen. Ther Adv Vaccin Immunother 2019;7:2515135518821623. 10.1177/2515135518821623 PMC6348545 30719508

[6] Damelin M , Geles KG, Follettie MT, Yuan P, Baxter M, Golas J, . Delineation of a cellular hierarchy in lung cancer reveals an oncofetal antigen expressed on tumor-initiating cells. Cancer Res 2011;71:4236–46. 21540235 10.1158/0008-5472.CAN-10-3919

[7] Kerk SA , Finkel KA, Pearson AT, Warner KA, Zhang Z, Nör F, . 5T4-targeted therapy ablates cancer stem cells and prevents recurrence of head and neck squamous cell carcinoma. Clin Cancer Res 2017;23:2516–27. 27780858 10.1158/1078-0432.CCR-16-1834 PMC5405006

[8] Wang R , Lai Q, Tang L, Tao Y, Yao Y, Liu Y, . A novel 5T4-targeting antibody-drug conjugate H6-DM4 exhibits potent therapeutic efficacy in gastrointestinal tumor xenograft models. Am J Cancer Res 2018;8:610–23. 29736307 PMC5934552

[9] Chuprakov S , Ogunkoya AO, Barfield RM, Bauzon M, Hickle C, Kim YC, . Tandem-cleavage linkers improve the in vivo stability and tolerability of antibody−drug conjugates. Bioconjug Chem 2021;32:746–54. 33689309 10.1021/acs.bioconjchem.1c00029

[10] Wei Q , Li P, Yang T, Zhu J, Sun L, Zhang Z, . The promise and challenges of combination therapies with antibody-drug conjugates in solid tumors. J Hematol Oncol 2024;17:1. 38178200 10.1186/s13045-023-01509-2 PMC10768262

[11] Dorywalska M , Dushin R, Moine L, Farias SE, Zhou D, Navaratnam T, . Molecular basis of valine-citrulline-PABC linker instability in site-specific ADCs and its mitigation by linker design. Mol Cancer Ther 2016;15:958–70. 26944918 10.1158/1535-7163.MCT-15-1004

[12] Drake PM , Albers AE, Baker J, Banas S, Barfield RM, Bhat AS, . Aldehyde tag coupled with HIPS chemistry enables the production of ADCs conjugated site-specifically to different antibody regions with distinct in vivo efficacy and PK outcomes. Bioconjug Chem 2014;25:1331–41. 24924618 10.1021/bc500189z PMC4215875

[13] Cao M , De Mel N, Jiao Y, Howard J, Parthemore C, Korman S, . Site-specific antibody-drug conjugate heterogeneity characterization and heterogeneity root cause analysis. MAbs 2019;6:1064–76. 10.1080/19420862.2019.1624127 PMC6748582 31198090

[14] Liu J , Barfield RM, Rabuka D. Site-specific bioconjugation using SMARTag^®^ technology: a practical and effective chemoenzymatic approach to generate antibody-drug conjugates. Methods Mol Biol 2019;2033:131–47. 31332752 10.1007/978-1-4939-9654-4_10

[15] Kantak S , Mendelsohn BA, Barfield RM, Chuprakov S, Drake PM, Ogunkoya A, inventors; Exelixis, Inc., assignee. 5t4 antibody-drug conjugates and uses thereof. WO Patent No. WO2023220620. 2023 November 16.

[16] Doronina SO , Toki BE, Torgov MY, Mendelsohn BA, Cerveny CG, Chace DF, . Development of potent monoclonal antibody auristatin conjugates for cancer therapy. Nat Biotechnol 2003;21:778–84. 12778055 10.1038/nbt832

[17] Haeckel A , Appler F, Ariza de Schellenberger A, Schellenberger E. XTEN as biological alternative to pegylation allows complete expression of a protease- activatable killin-based cytostatic. PLoS One 2016;11:e0157193. 27295081 10.1371/journal.pone.0157193 PMC4905650

[18] Viricel W , Fournet G, Beaumel S, Perrial E, Papot S, Dumontet C, . Monodisperse polysarcosine-based highly-loaded antibody-drug conjugates. Chem Sci 2019;10:4048–53. 31015945 10.1039/c9sc00285e PMC6457330

[19] Giese M , Davis PD, Woodman RH, Hermanson G, Pokora A, Vermillion M. Linker architectures as steric auxiliaries for altering enzyme-mediated payload release from bioconjugates. Bioconjug Chem 2021;32:2257–67. 34587447 10.1021/acs.bioconjchem.1c00429

[20] Evans N , Grygorash R, Williams P, Kyle A, Kantner T, Pathak R, . Incorporation of hydrophilic macrocycles into drug-linker reagents produces antibody-drug conjugates with enhanced in vivo performance. Front Pharmacol 2022;13:764540. 35784686 10.3389/fphar.2022.764540 PMC9247464

[21] Toader D , Fessler SP, Collins SD, Conlon PR, Bollu R, Catcott KC, . Discovery and preclinical characterization of XMT-1660, an optimized B7-H4-targeted antibody-drug conjugate for the treatment of cancer. Mol Cancer Ther 2023;22:999–1012. 37294948 10.1158/1535-7163.MCT-22-0786 PMC10477829

[22] Watanabe T , Arashida N, Fujii T, Shikida N, Ito K, Shimbo K, . Exo-cleavable linkers: enhanced stability and therapeutic efficacy in antibody–drug conjugates. J Med Chem 2024;67:18124–38. 39410752 10.1021/acs.jmedchem.4c01251 PMC11513888

[23] Kudirka R , Barfield RM, McFarland J, Albers AE, de Hart GW, Drake PM, . Generating site-specifically modified proteins via a versatile and stable nucleophilic carbon ligation. Chem Biol 2015;22:293–8. 25619935 10.1016/j.chembiol.2014.11.019

[24] Shapiro GI , Vaishampayan UN, LoRusso P, Barton J, Hua S, Reich SD, . First-in-human trial of an anti-5T4 antibody-monomethylauristatin conjugate, PF-06263507, in patients with advanced solid tumors. Invest New Drugs 2017;35:315–23. 28070718 10.1007/s10637-016-0419-7 PMC5418317

[25] Junutula JR , Raab H, Clark S, Bhakta S, Leipold D, Weir S, . Site-specific conjugation of a cytotoxic drug to an antibody improves the therapeutic index. Nat Biotechnol 2008;26:925–32. 18641636 10.1038/nbt.1480

[26] Strop P , Liu S-H, Dorywalska M, Delaria K, Dushin RG, Tran TT, . Location matters: site of conjugation modulates stability and pharmacokinetics of antibody drug conjugates. Chem Biol 2013;20:161–7. 23438745 10.1016/j.chembiol.2013.01.010

[27] Smith RA , Zammit DJ, Damle NK, Usansky H, Reddy SP, Lin J-H, . ASN004, A 5T4-targeting scFv-Fc antibody–drug conjugate with high drug-to-antibody ratio, induces complete and durable tumor regressions in preclinical models. Mol Cancer Ther 2021;20:1327–37. 34045226 10.1158/1535-7163.MCT-20-0565

[28] Eaton JS , Miller PE, Mannis MJ, Murphy CJ. Ocular adverse events associated with antibody-drug conjugates in human clinical trials. J Ocul Pharmacol Ther 2015;10:589–604. 10.1089/jop.2015.0064 PMC4677113 26539624

[29] Donaghy H . Effects of antibody, drug and linker on the preclinical and clinical toxicities of antibody-drug conjugates. MAbs 2016;8:659–71. 27045800 10.1080/19420862.2016.1156829 PMC4966843

[30] Coleman RL , Lorusso D, Gennigens C, González-Martín A, Randall L, Cibula D, . Efficacy and safety of tisotumab vedotin in previously treated recurrent or metastatic cervical cancer (innovaTV 204/GOG-3023/ENGOT-cx6): a multicentre, open-label, single-arm, phase 2 study. Lancet Oncol 2021;22:609–19. 33845034 10.1016/S1470-2045(21)00056-5

[31] Arn CR , Halla KJ, Gill S. Tisotumab vedotin safety and tolerability in clinical practice: managing adverse events. J Adv Pract Oncol 2023;14:139–52. 37009403 10.6004/jadpro.2023.14.2.4 PMC10062530

[32] Maecker H , Jonnalagadda V, Bhakta S, Jammalamadaka V, Junutula JR. Exploration of the antibody–drug conjugate clinical landscape. Mabs 2023;15:2229101. 37639687 10.1080/19420862.2023.2229101 PMC10464553

