# Supplementary material for: Preclinical Characterization of XB010: A Novel Antibody–Drug Conjugate for the Treatment of Solid Tumors that Targets Tumor-Associated Antigen 5T4
Source: Mol Cancer Ther. 2025 Aug 21;24(12):1856–66. doi: 10.1158/1535-7163.MCT-24-1014 (PMC12670076; doi:10.1158/1535-7163.MCT-24-1014)
Supplement: Figure S1 — SEC-HPLC chromatograms of EXMA-001 and EXMA-004. A single monomeric peak was observed for EXMA-001 and EXMA-004, indicating promising developability. [file mct-24-1014_figure_s1_suppsf1.docx]

**Figure S1.** SEC-HPLC chromatograms.

**
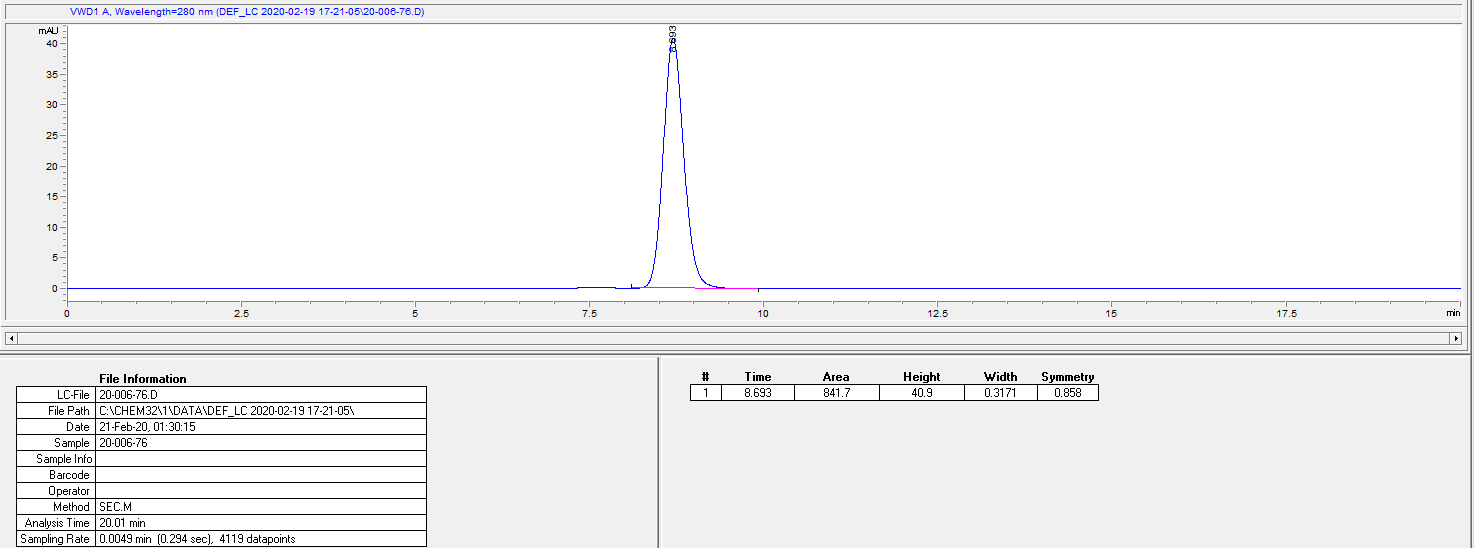
A**

*
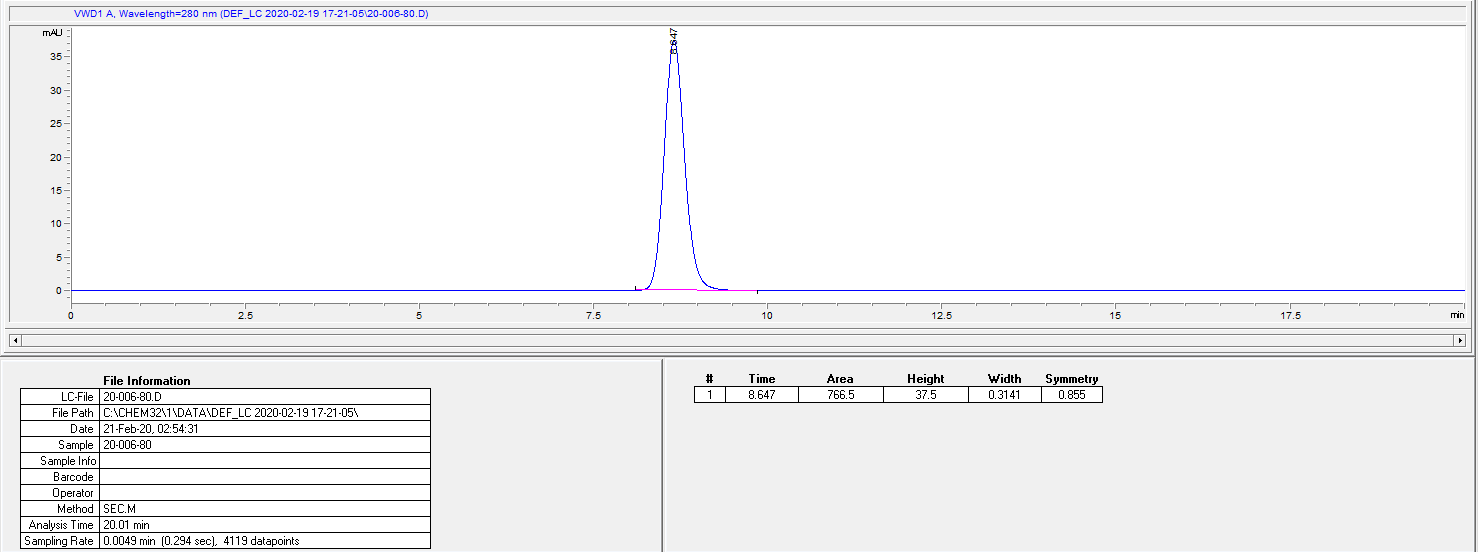
***B**

A single monomeric peak was observed for (**A**) EXMA-001 and (**B**) EXMA-004, indicating promising developability.

SEC-HPLC, size exclusion high performance liquid chromatography
