# Supplementary material for: Preclinical Characterization of XB010: A Novel Antibody–Drug Conjugate for the Treatment of Solid Tumors that Targets Tumor-Associated Antigen 5T4
Source: Mol Cancer Ther. 2025 Aug 21;24(12):1856–66. doi: 10.1158/1535-7163.MCT-24-1014 (PMC12670076; doi:10.1158/1535-7163.MCT-24-1014)
Supplement: Figure S2 — Antigen binding of EXMA-001 and EXMA-004. Oxidative stress testing of EXMA-001 and EXMA-004 showed no changes in antigen binding after treatment with hydrogen peroxide. By comparison, an oxidation event taking place in the complementarity-determining region of the antibody altered the binding of EXMA-005 to the target antigen, meaning that this candidate was not considered suitable for further development. [file mct-24-1014_figure_s2_suppsf2.docx]

**Figure S2.** Antigen binding of EXMA-001 and EXMA-004.


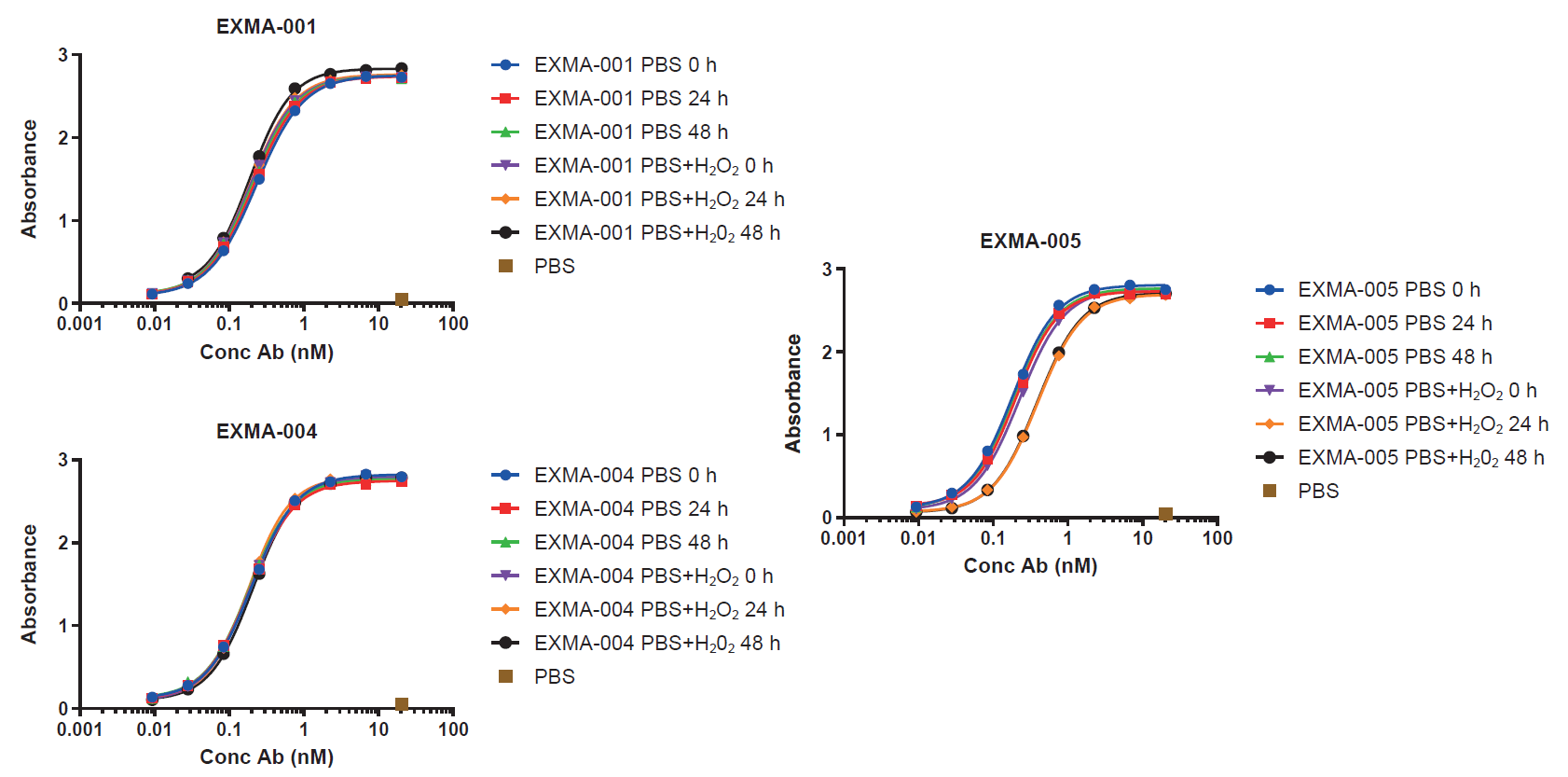


Oxidative stress testing of EXMA-001 and EXMA-004 (left) showed no changes in antigen binding after treatment with hydrogen peroxide. By comparison, in the example shown on the right (EXMA-005), an oxidation event taking place in the complementarity-determining region of the antibody altered its binding to the target antigen, meaning that this candidate was not considered suitable for further development.
Ab, antibody; Conc, concentration; H_2_O_2_, hydrogen peroxide; PBS, phosphate buffered saline.
