# Supplementary material for: Preclinical Characterization of XB010: A Novel Antibody–Drug Conjugate for the Treatment of Solid Tumors that Targets Tumor-Associated Antigen 5T4
Source: Mol Cancer Ther. 2025 Aug 21;24(12):1856–66. doi: 10.1158/1535-7163.MCT-24-1014 (PMC12670076; doi:10.1158/1535-7163.MCT-24-1014)
Supplement: Figure S3 — Structure of XB010. Chemical structure of the linker (RED-601) and monomethyl auristatin E payload, conjugated to the EXMA-001-ST monoclonal antibody using site-specific SMARTag technology. [file mct-24-1014_figure_s3_suppsf3.docx]

**Figure S3.** Structure of XB010

Chemical structure of the linker (RED-601) and monomethyl auristatin E payload, conjugated to the EXMA-001-ST monoclonal antibody using site-specific SMARTag® technology.
