# Supplementary material for: Preclinical Characterization of XB010: A Novel Antibody–Drug Conjugate for the Treatment of Solid Tumors that Targets Tumor-Associated Antigen 5T4
Source: Mol Cancer Ther. 2025 Aug 21;24(12):1856–66. doi: 10.1158/1535-7163.MCT-24-1014 (PMC12670076; doi:10.1158/1535-7163.MCT-24-1014)
Supplement: Figure S4 — HIC analysis of XB010. The DAR was confirmed as 1.8, as measured using peak areas from HIC versus a reference standard. [file mct-24-1014_figure_s4_suppsf4.docx]

**Figure S4.** HIC analysis of XB010.


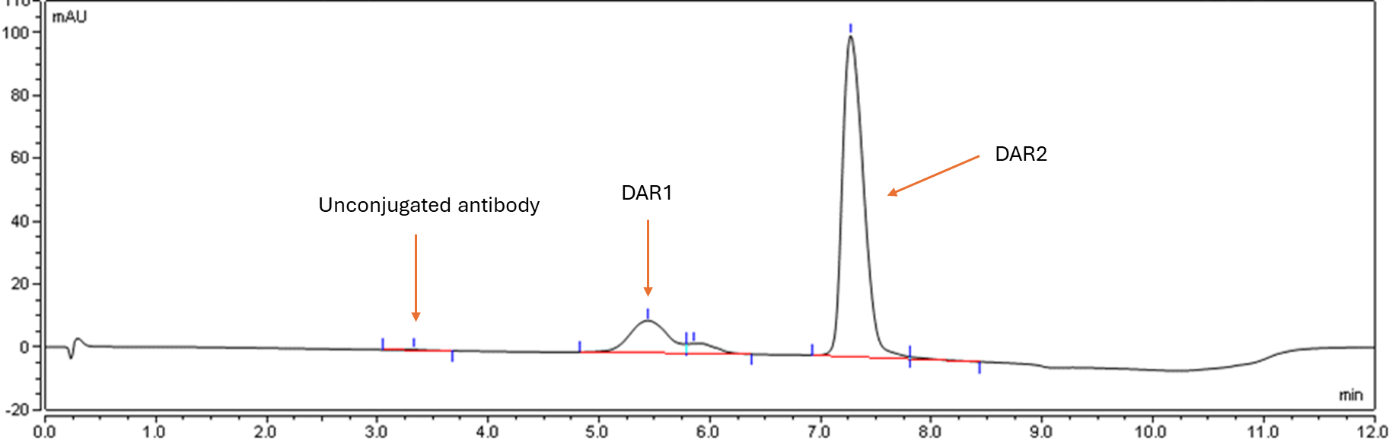


The DAR was confirmed as 1.8, as measured using peak areas from HIC versus a reference standard.

DAR, drug-to-antibody ratio; HIC, hydrophobic interaction chromatography.
