# Supplementary material for: Preclinical Characterization of XB010: A Novel Antibody–Drug Conjugate for the Treatment of Solid Tumors that Targets Tumor-Associated Antigen 5T4
Source: Mol Cancer Ther. 2025 Aug 21;24(12):1856–66. doi: 10.1158/1535-7163.MCT-24-1014 (PMC12670076; doi:10.1158/1535-7163.MCT-24-1014)
Supplement: Figure S5 — RP-HPLC analysis of XB010 following single and tandem enzymatic cleavage. Single and tandem enzymatic cleavage with β-glucuronidase and cathepsin B confirmed the release of unconjugated MMAE. [file mct-24-1014_figure_s5_suppsf5.docx]

**Figure S5.** RP-HPLC analysis of XB010 following single and tandem enzymatic cleavage.


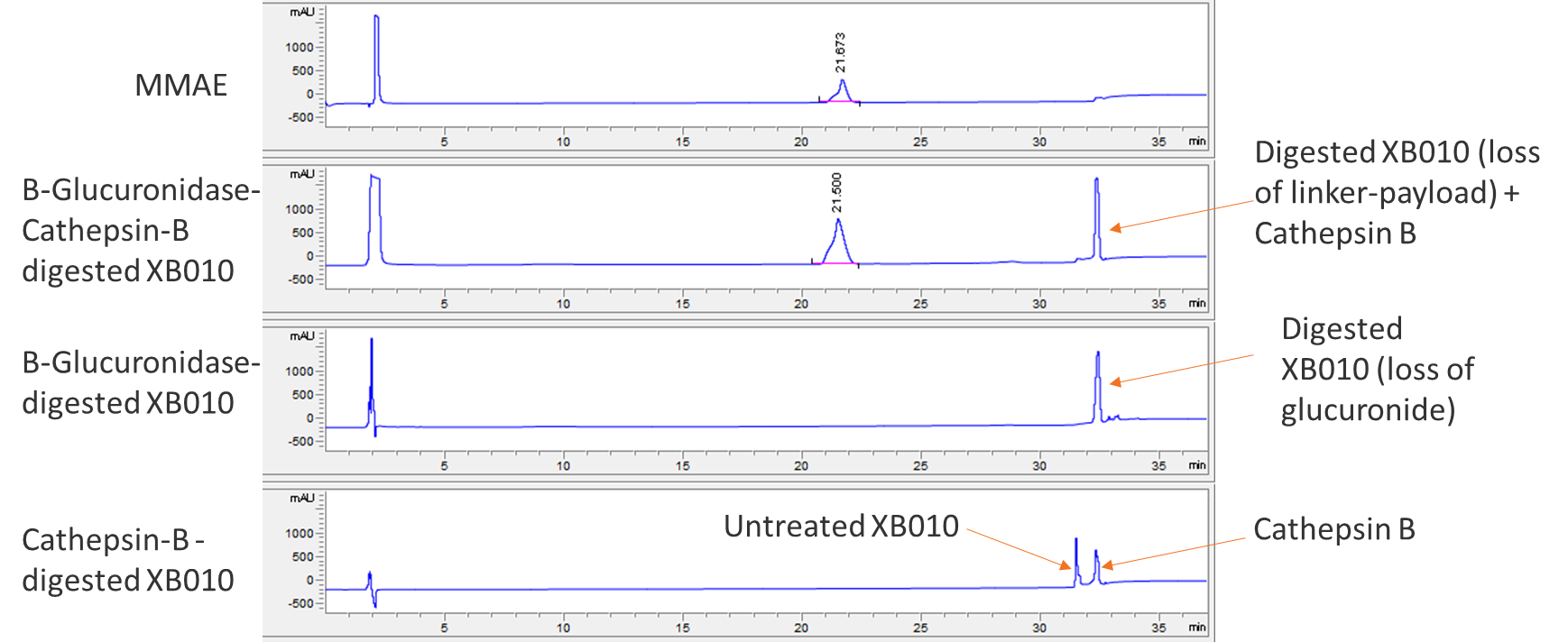
Single and tandem enzymatic cleavage with β-glucuronidase and cathepsin B confirmed the release of unconjugated MMAE. Material released from XB010 exhibited the same retention time as MMAE.

MMAE, monomethyl auristatin E.
