# Supplementary material for: Preclinical Characterization of XB010: A Novel Antibody–Drug Conjugate for the Treatment of Solid Tumors that Targets Tumor-Associated Antigen 5T4
Source: Mol Cancer Ther. 2025 Aug 21;24(12):1856–66. doi: 10.1158/1535-7163.MCT-24-1014 (PMC12670076; doi:10.1158/1535-7163.MCT-24-1014)
Supplement: Figure S6 — Translocation of XB010 to endocytic compartments in MCF-7 cells, MCF-7 cell confluency over time with XB010, and quantification of XB010 in MCF-7 LAMP-1 cell compartments after 1 and 6 hours of incubation. [file mct-24-1014_figure_s6_suppsf6.docx]

**Figure S6.** (**A**) and (**B**) translocation of XB010 to endocytic compartments in MCF-7 cells, (**C**) MCF-7 cell confluency over time with XB010, and (**D**) quantification of XB010 in MCF-7 LAMP-1 cell compartments after 1 and 6 hours of incubation.


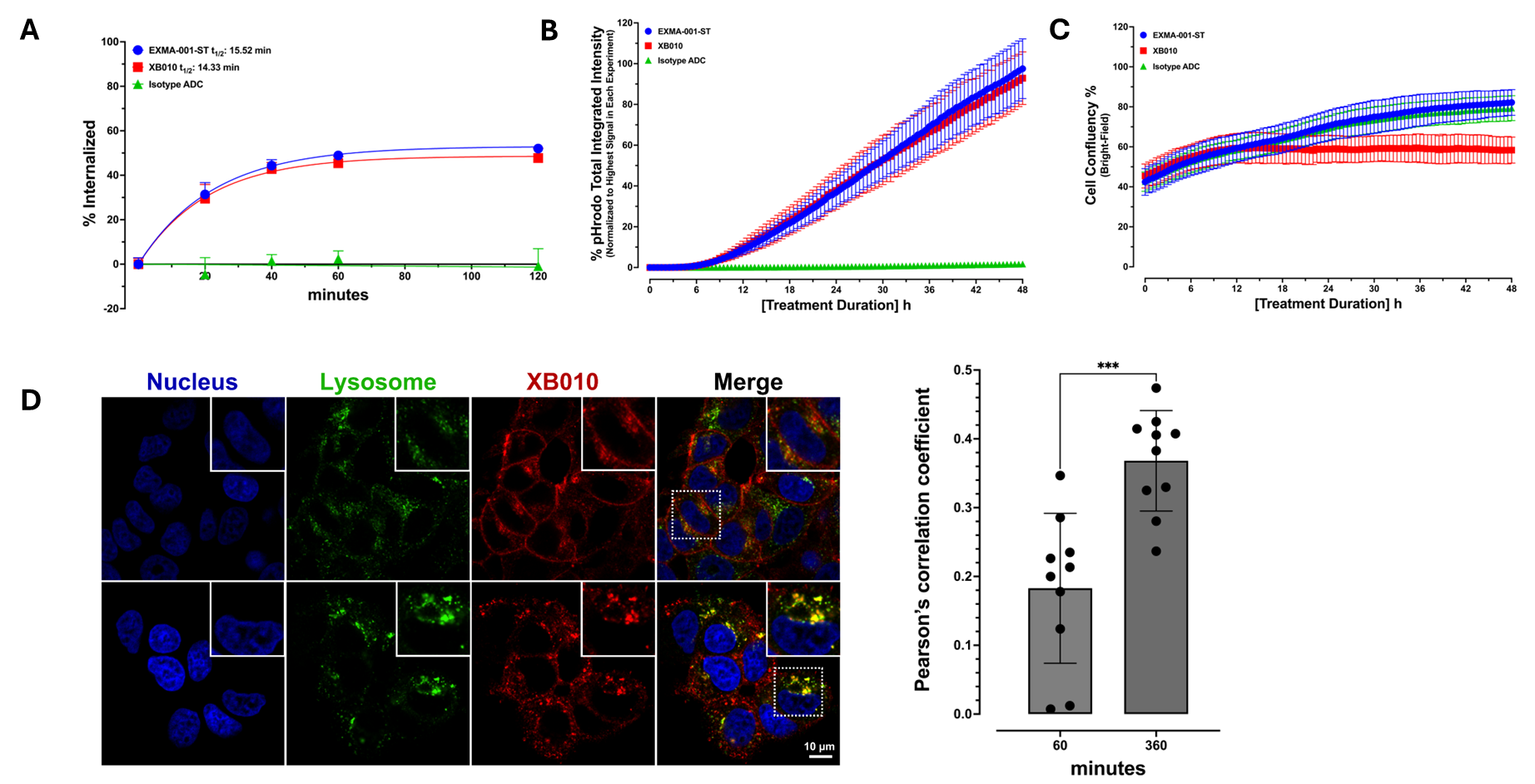


****P*<0.001 (unpaired t-test). Data presented are mean ± SEM. ADC, antibody-drug conjugate; SEM, standard error of the mean;
t_½_, half-life.
