# Supplementary material for: Preclinical Characterization of XB010: A Novel Antibody–Drug Conjugate for the Treatment of Solid Tumors that Targets Tumor-Associated Antigen 5T4
Source: Mol Cancer Ther. 2025 Aug 21;24(12):1856–66. doi: 10.1158/1535-7163.MCT-24-1014 (PMC12670076; doi:10.1158/1535-7163.MCT-24-1014)
Supplement: Figure S7 — TK profile of XB010 in rats. Following single IV doses of XB010 (30, 60, and 90 mg/kg) administered to female Sprague Dawley rats, linear TK profiles were observed over 11 days. Near-identical TK profiles were observed for the total antibody and total ADC, demonstrating the stability of XB010. [file mct-24-1014_figure_s7_suppsf7.docx]

**Figure S7.** TK profile of XB010 in rats.


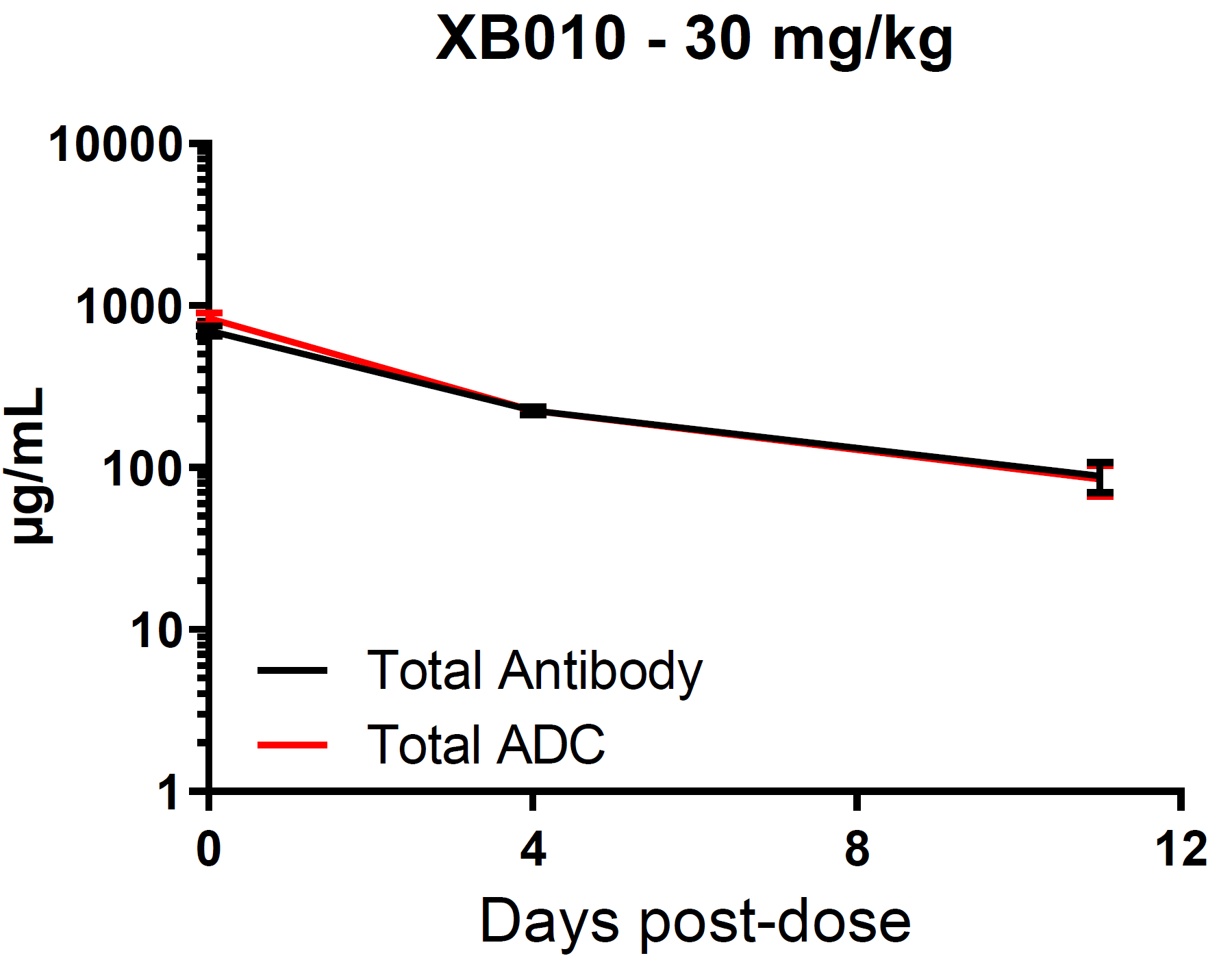

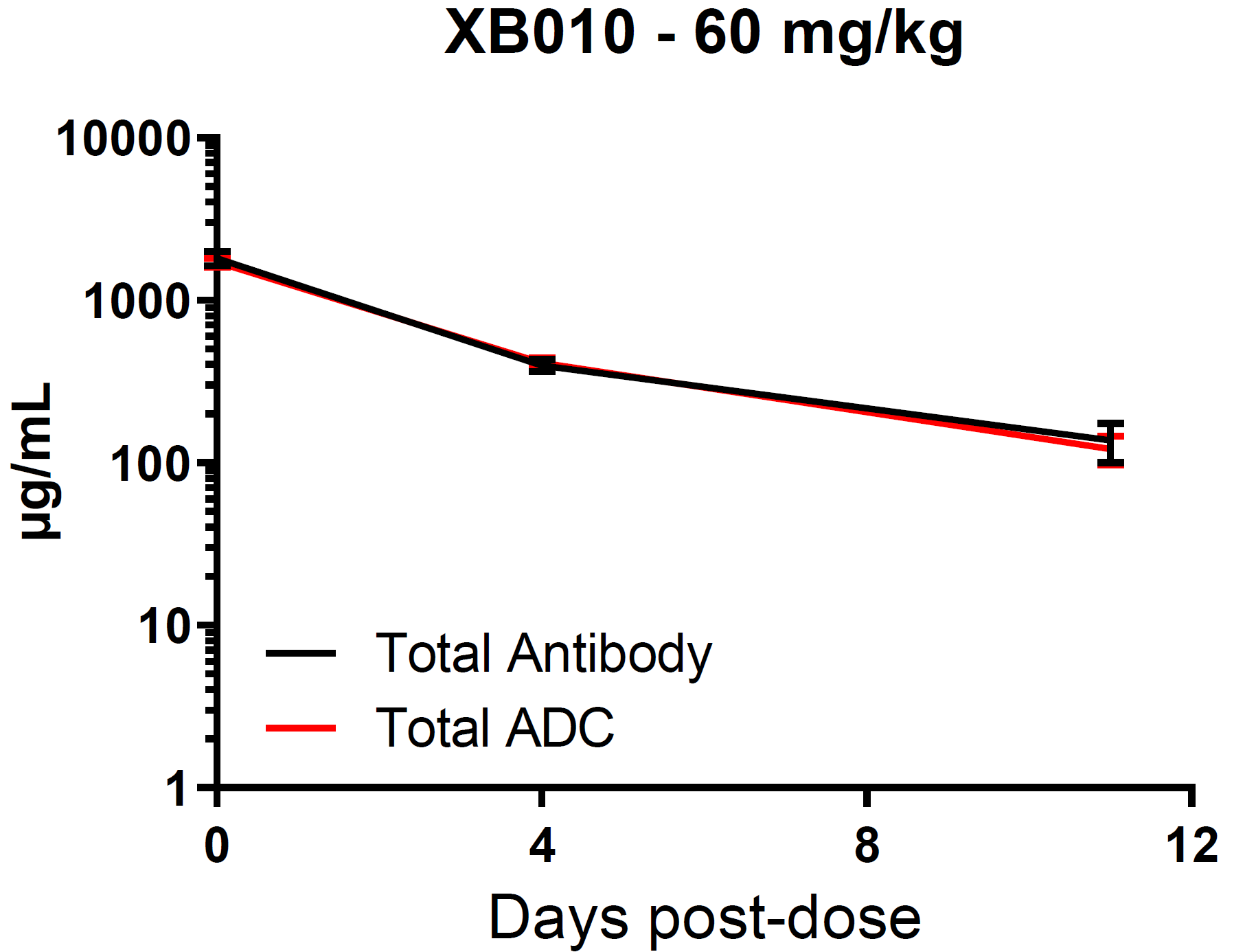


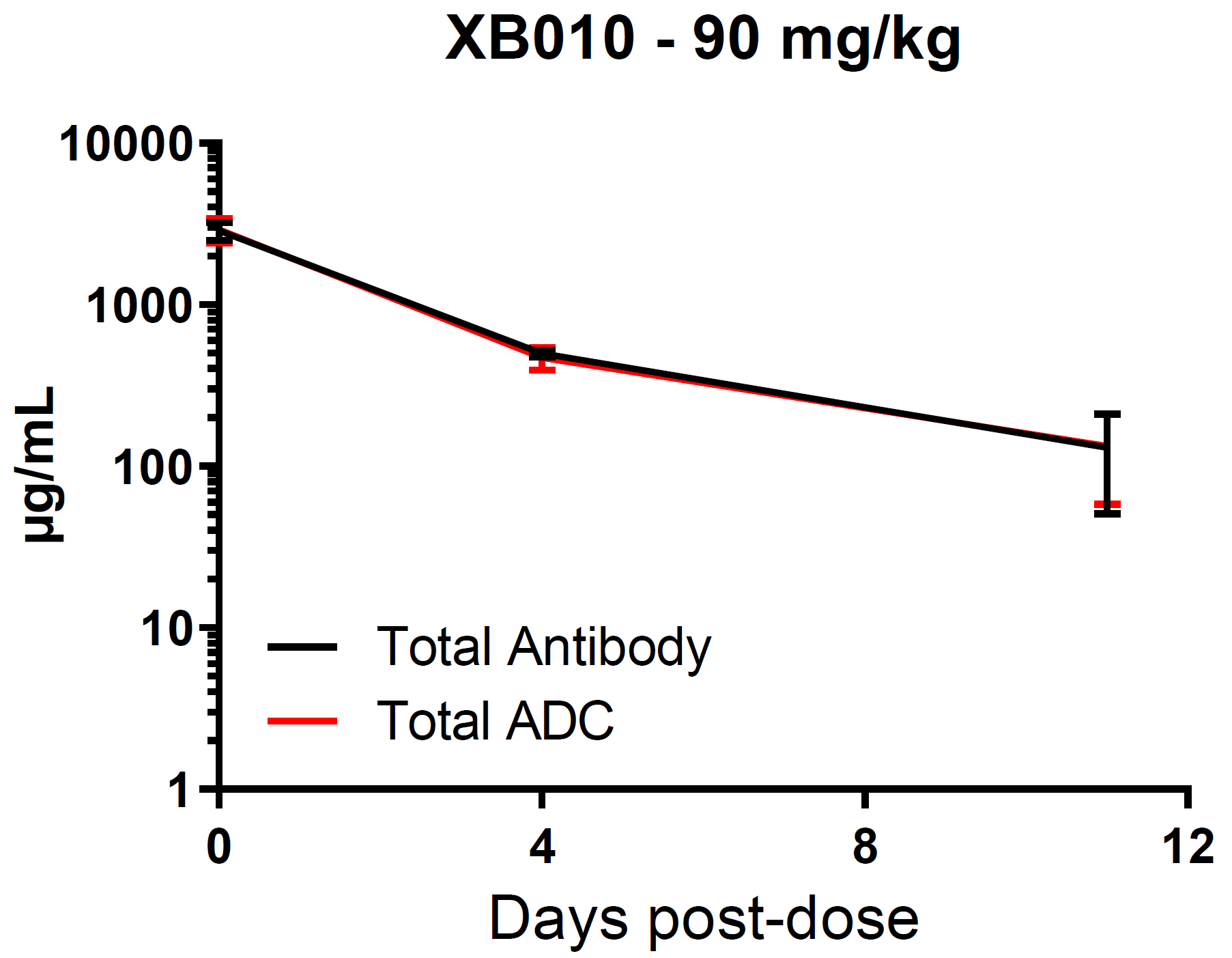


Following single IV doses of XB010 (30, 60, and 90 mg/kg) administered to female Sprague Dawley rats, linear TK profiles were observed over 11 days. Near-identical TK profiles were observed for the total antibody and total ADC, demonstrating the stability of XB010.

ADC, antibody-drug conjugate; IV, intravenous; TK, toxicokinetic.
