# Supplementary material for: Preclinical Characterization of XB010: A Novel Antibody–Drug Conjugate for the Treatment of Solid Tumors that Targets Tumor-Associated Antigen 5T4
Source: Mol Cancer Ther. 2025 Aug 21;24(12):1856–66. doi: 10.1158/1535-7163.MCT-24-1014 (PMC12670076; doi:10.1158/1535-7163.MCT-24-1014)
Supplement: Figure S8 — TK profile of XB010 in NHPs. Following repeat IV doses of XB010 (1, 6, and 25 mg/kg) administered to NHPs on days 1 and 22, non-linear TK profiles were observed, indicating target-mediated drug disposition. XB010 was stable in NHPs, with nearly identical TK profiles for the total antibody and total ADC, and low levels of unconjugated MMAE. [file mct-24-1014_figure_s8_suppsf8.docx]

**Figure S8.** TK profile of XB010 in NHPs.


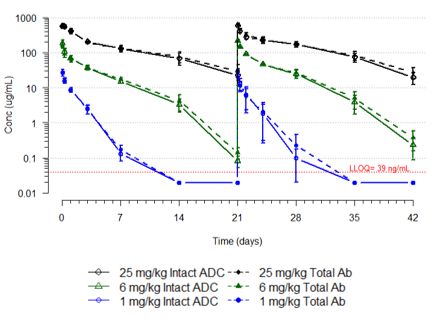


Following repeat IV doses of XB010 (1, 6, and 25 mg/kg) administered to NHPs on days 1 and 22, non-linear TK profiles were observed, indicating target-mediated drug disposition. XB010 was stable in NHPs, with nearly identical TK profiles for the total antibody and total ADC, and low levels of unconjugated MMAE.

ADC, antibody-drug conjugate; IV, intravenous; MMAE, monomethyl auristatin E; NHP, non-human primate; TK, toxicokinetic.
