# Supplementary material for: Preclinical Characterization of XB010: A Novel Antibody–Drug Conjugate for the Treatment of Solid Tumors that Targets Tumor-Associated Antigen 5T4
Source: Mol Cancer Ther. 2025 Aug 21;24(12):1856–66. doi: 10.1158/1535-7163.MCT-24-1014 (PMC12670076; doi:10.1158/1535-7163.MCT-24-1014)
Supplement: Table S1 — Summary of lead mAb and ADC properties. [file mct-24-1014_table_s1_suppst1.docx]

**Table S1.** Summary of lead mAb and ADC properties (EXMA-001, EXMA-004, and XB010).

| **Lead mAb** | **Sequence liabilities (CDR)** | **Thermal stability (DSF; *T_m_*, °C)** | **Cross-species reactivity**  **(Binding to 5T4-CHO cells; flow cytometry)** | | | | **Monovalent affinity**  **(SPR; K_D_, nM)** | | **Internalization**  **(live cell imaging; MFI vs benchmark)** | |
| --- | --- | --- | --- | --- | --- | --- | --- | --- | --- | --- |
|  |  |  | **Human 5T4** | **Cyno  5T4** | **Mouse 5T4** | **Rat  5T4** | **Human 5T4** | **Cyno  5T4** | **Hu-5T4-HER293** | **MCF-7** |
| EXMA-001 | None | 86.4 | Yes | Yes | No | No | 8.8 | 27.5 | +++ | +++ |
| EXMA-004 | None | 81.9 | Yes | Yes | No | No | 28.9 | 32.5 | +++ | +++ |
| XB010 |  |  |  |  |  |  | 7.5 | 26.3 |  |  |

ADC, antibody-drug conjugate; CDR, complementary-determining region; CHO, Chinese hamster ovary; Cyno, cynomolgus monkey; DSF, differential scanning fluorimetry; mAb, monoclonal antibody; MFI, mean fluorescence intensity; SPR, surface plasmon resonance.
Grey shading indicates experiments not performed
