# Supplementary material for: Preclinical Characterization of XB010: A Novel Antibody–Drug Conjugate for the Treatment of Solid Tumors that Targets Tumor-Associated Antigen 5T4
Source: Mol Cancer Ther. 2025 Aug 21;24(12):1856–66. doi: 10.1158/1535-7163.MCT-24-1014 (PMC12670076; doi:10.1158/1535-7163.MCT-24-1014)
Supplement: Table S2 — Tumor growth inhibition, response rates, and survival following IV administration of XB010 and controls (single dose in MCF-7 xenograft model). [file mct-24-1014_table_s2_suppst2.docx]

**Table S2.** Tumor growth inhibition, response rates, and survival following IV administration of XB010 and controls (single dose in MCF-7 xenograft model).

| **Group** | **CR** | **%TGI vs vehicle (day 26)** | ***P*-value vs vehicle^a^** | **Median survival**  **(days)** | **Log-rank test**  **(Mantel-Cox)**  ***P*-value vs vehicle** |
| --- | --- | --- | --- | --- | --- |
| Vehicle | 0 | -- | -- | 29.5 | -- |
| Isotype ADC,  5 mg/kg | 0 | 4.9 | NS | 31 | NS |
| 5T4 targeting mafodotin-ADC, 5 mg/kg | 1 | 80 | <0.001 | >58 | <0.001 |
| XB010, 0.5 mg/kg | 0 | 11 | NS | 29 | NS |
| XB010, 2.5 mg/kg | 2 | 50 | <0.05 | 47 | <0.05 |
| XB010, 5 mg/kg | 6 | 93 | <0.0001 | >58 | <0.0001 |

^a^Statistical differences for %TGI vs vehicle were determined using one-way ANOVA with Dunnett's *post hoc* test, as appropriate.

ADC, antibody-drug conjugate; ANOVA, analysis of variance; CR, complete response (defined as a tumor volume of 0 mm^3^ for three consecutive measurements); IV, intravenous; NS, *P*>0.05; %TGI, percent tumor growth inhibition (defined as [1-Ti/Ci] × 100, where Ti and Ci are the mean tumor volumes of the treatment and control groups, respectively, on a given day).
