# Supplementary material for: Preclinical Characterization of XB010: A Novel Antibody–Drug Conjugate for the Treatment of Solid Tumors that Targets Tumor-Associated Antigen 5T4
Source: Mol Cancer Ther. 2025 Aug 21;24(12):1856–66. doi: 10.1158/1535-7163.MCT-24-1014 (PMC12670076; doi:10.1158/1535-7163.MCT-24-1014)
Supplement: Table S5 — Tumor growth inhibition, response rates, and survival following IV administration of XB010 alone or in combination with anti-PD1 (h5T4-MC38 model). [file mct-24-1014_table_s5_suppst5.docx]

**Table S5.** Tumor growth inhibition, response rates, and survival following IV administration of XB010 alone or in combination with anti-PD1 (h5T4-MC38 model).

| **Group** | **Dose level (mg/kg)** | **CR** | **%TGI vs vehicle (day 18)** | ***P*-value^a^ vs vehicle** | **Median survival**  **(days)** | **Log-rank test**  **(Mantel-Cox)**  ***P*-value vs vehicle** |
| --- | --- | --- | --- | --- | --- | --- |
| Vehicle (PBS)  Vehicle (XB010) | - | 0 | -- | -- | 22 | -- |
|  | - |  |  |  |  |  |
| Vehicle (PBS)  XB010 | - | 4 | 71 | <0.001 | 39 | <0.001 |
|  | 5 |  |  |  |  |  |
| Anti-PD1  Vehicle (XB010) | 0.75 | 0 | 55 | NS | 29 | <0.001 |
|  | - |  |  |  |  |  |
| Anti-PD1  Vehicle (XB010) | 0.5 | 0 | 57 | NS | 30.5 | <0.001 |
|  | - |  |  |  |  |  |
| Anti-PD1  XB010 | 0.75 | 9 | 97 | <0.001 | >55 | <0.001 |
|  | 5 |  |  |  |  |  |
| Anti-PD1  XB010 | 0.5 | 5 | 96 | <0.001 | >55 | <0.001 |
|  | 5 |  |  |  |  |  |

^a^Statistical differences for %TGI vs vehicle were determined using Kruskal–Wallis ANOVA with Conover's *post hoc* test.

ANOVA, analysis of variance; CR, complete response (defined as a tumor volume of 0 mm^3^ for three consecutive measurements); IV, intravenous; NS, *P*>0.05; PBS, phosphate buffered saline; PD-1, programmed cell death protein 1; %TGI, percent tumor growth inhibition, defined as (1-(Vt/V0) / (Ct-C0)) × 100 on the day indicated, where V was the treated tumor volume at time t or 0, and C was the control (vehicle) volume).
