# Supplementary material for: Preclinical Characterization of XB010: A Novel Antibody–Drug Conjugate for the Treatment of Solid Tumors that Targets Tumor-Associated Antigen 5T4
Source: Mol Cancer Ther. 2025 Aug 21;24(12):1856–66. doi: 10.1158/1535-7163.MCT-24-1014 (PMC12670076; doi:10.1158/1535-7163.MCT-24-1014)
Supplement: Table S6 — Mean TK parameters for XB010 (total ADC) administered following single-dose administration to rats over an 11-day observation period. [file mct-24-1014_table_s6_suppst6.docx]

**Table S6***.* Mean TK parameters for XB010 (total ADC) administered following single-dose administration to rats over an 11-day observation period.

| **Dose**  **(mg/kg)** | **C_max_**  **(µg/mL)** | **AUC_last_**  **(µg.day/mL)** |
| --- | --- | --- |
| 30 | 833.7 (71.1) | 2858.9  (23.8) |
| 60 | 1717.1 (113.5) | 5315.3  (293.4) |
| 90^a^ | 2906.8 (499) | 7590.1 (1007.1) |

Data are presented as mean (SD) of n=5/group.

^a^Data from two animals were excluded due to missing samples or less than the lower limit of quantification.
ADC, antibody-drug conjugate; AUC_last_, area under the concentration-time curve (from time 0 to last observation); C_max_, maximum plasma concentration; TK, toxicokinetic.
