# Supplementary material for: Preclinical Characterization of XB010: A Novel Antibody–Drug Conjugate for the Treatment of Solid Tumors that Targets Tumor-Associated Antigen 5T4
Source: Mol Cancer Ther. 2025 Aug 21;24(12):1856–66. doi: 10.1158/1535-7163.MCT-24-1014 (PMC12670076; doi:10.1158/1535-7163.MCT-24-1014)
Supplement: Table S7 — Toxicity of XB010 (30, 60 and 90 mg/kg doses) in rats. [file mct-24-1014_table_s7_suppst7.docx]

**Table S7.** Toxicity of XB010 (30, 60 and 90 mg/kg doses) in rats.

| **Test article** | **Dose (mg/kg)** | **N** | **Tolerated?  (% mortality)** |
| --- | --- | --- | --- |
| Vehicle control | – | 5 | Yes (0%) |
| XB010 | 30 | 5 | Yes (0%) |
|  | 60 | 5 | Yes (0%) |
|  | 90 | 5 | No (40%) |
