# Supplementary material for: Preclinical Characterization of XB010: A Novel Antibody–Drug Conjugate for the Treatment of Solid Tumors that Targets Tumor-Associated Antigen 5T4
Source: Mol Cancer Ther. 2025 Aug 21;24(12):1856–66. doi: 10.1158/1535-7163.MCT-24-1014 (PMC12670076; doi:10.1158/1535-7163.MCT-24-1014)
Supplement: Table S8 — Toxicity of XB010 (1, 6 and 25 mg/kg doses) in NHPs. [file mct-24-1014_table_s8_suppst8.docx]

**Table S8.** Toxicity of XB010 (1, 6 and 25 mg/kg doses) in NHPs.

| **Test article** | **Dose (mg/kg)** | **N** | **Tolerated?** |
| --- | --- | --- | --- |
| Vehicle control | – | 3 | Yes |
| XB010 | 1 | 3 | Yes |
|  | 6 | 3 | Yes |
|  | 25 | 3 | Yes |

NHP, non-human primate.
